# FDA preemption of conflicting state drug regulation and the looming battle over abortion medications

**DOI:** 10.1093/jlb/lsad005

**Published:** 2023-03-15

**Authors:** Peter Grossi, Daphne O’Connor

**Keywords:** FDA preemption, mifepristone, Mifeprex, abortion, REMS, FDCA, *Levine*, *Mensing*, *Bartlett*, *Albrecht*, *Dobbs*, abortion pills

## Abstract

Over the past 25 years, Congress and the FDA have determined the safest and most beneficial way to regulate the use of mifepristone (Mifeprex), the medication that accounts for the majority of abortions in the United States. The *Dobbs* decision has renewed the importance of those scientific determinations, especially FDA's decisions implementing the Risk Evaluation and Mitigation Strategy (REMS) provisions of the Federal Food Drug and Cosmetic Act (FDCA), that mifepristone may be taken by patients outside the presence of any healthcare provider (often through telemedicine prescription and shipment across state lines). Now that *Dobbs* has been decided, state officials have indicated that they will seek to enforce state statutes which conflict with FDA's regimen for the proper use of mifepristone, by banning its use entirely, prohibiting telemedicine prescription, or imposing other requirements which FDA has specifically considered and now rejected as contrary to the congressional mandate that FDA-approved drugs be as accessible as safety considerations allow. Litigation has already been filed to invalidate such statutes on the grounds that they are preempted by the doctrine that state law which conflicts with, or undermines the purposes of, FDA actions with respect to approved drugs are preempted under the Supremacy Clause of the Constitution. This article examines the Supreme Court caselaw and FDA actions which will dictate the outcome of that litigation. Part I details the statutory basis for FDA preemption of conflicting state law and the four decisions by the Supreme Court over the last 13 years (*Levine, Mensing, Bartlett* and *Albrecht*) which enunciate the governing legal standards for FDA preemption. We pay particular attention to the opinions of Justice Alito and the other conservative justices, which hold that such FDA preemption should be robust to ensure that there is one consistent, national policy for the distribution and regulation of drugs, under the science-based decisions of the FDA, rather than the 'parochialism' of differing state standards. Part II details FDA's comprehensive program for the balanced, though appropriately restricted, use of mifepristone, and the 22 years of FDA actions that brought that about. It then catalogs the state statutes limiting the use of the drug which, in material ways, conflict with those FDA determinations. Part III outlines the arguments made in one early lawsuit seeking preemption of the statutes of one state (Mississippi)–a law suit which previews the wider litigation to come. It then sets forth the strong arguments for FDA preemption of each type of state restriction and responses to the 'defenses' of those statutes that have been offered in an effort to avoid FDA preemption under the Supremacy Clause. That review shows that a straight-forward application of the FDCA and the Supreme Court caselaw should result in the preemption of the state restrictions that squarely conflict with the relatively free access to abortion medications which FDA has mandated.

The *Dobbs* decision^1^ has focused legislators, administrative officials, health care providers, and advocates for both sides in the abortion debate on a body of law that previously had been of interest to only a devoted cadre of drug manufacturers and the lawyers who litigate the safety of their products—the doctrine that FDA’s actions pursuant to the Federal Food, Drug and Cosmetic Act (FDCA) preempt conflicting state regulation by virtue of the Supremacy Clause.^2^ Now that that wider audience has had some time to explore that preemption jurisprudence, those who favor unrestricted access to abortion medications—including the Biden Administration—see FDA preemption as a bulwark against state restrictions on the free flow of such drugs, which already account for a majority of all abortion procedures.^3^ Within hours of the *Dobbs* decision, Attorney General Garland reminded all concerned that ‘the FDA has approved the use of the medication Mifepristone’, the primary abortion drug, and ‘[s]tates may not ban Mifepristone based on disagreement with the FDA’s expert judgment about its safety and efficacy’.^4^

On the other hand, those who oppose abortions entirely—or who would restrict them significantly—view such FDA preemption as a threat to the states’ rights victory they believe they achieved in *Dobbs*. They argue, in litigation that has already begun, that abortion medications such as mifepristone are not entitled to the same protection afforded other FDA-approved drugs. And the cases to come are likely to center on the distribution of those medications by ‘telehealth’ providers across state lines—a practice that will raise conflicts not only between FDA regulation and state restrictions, but among conflicting state laws as well.^5^

In their zeal, both sides in the abortion debate tend to ignore the specific facts at issue in the Supreme Court’s four attempts to set forth a coherent set of principles applying that doctrine. Yet, as every member of the Court has stated on one or more occasions—especially in their most recent decision (*Albrecht*)—this is one of those legal doctrines where ‘Facts Matter’.

For example, in a recent article in the Liberty University Law Review, the Senior Counsel of Americans United for Life and the CEO of the American Association of Pro-Life Obstetricians and Gynecologists (‘AAPLOG’) recount their efforts over the past 20 years first to prevent FDA from approving mifepristone at all, and then to convince the Agency to restrict its use.^6^ Although they were unsuccessful at the federal level, the pro-life organizations were able to convince many legislatures to impose significant restrictions at the state level.^7^

The Liberty Law article argues that those restrictions should now be enacted in every state. And the authors assert, on the basis of a single introductory remark in the Supreme Court’s 2009 decision in *Wyeth v Levine*,^8^ that FDA preemption should not be any impediment to that effort.^9^ But the authors do not discuss the facts or actual holding of *Levine*; nor do they mention the three subsequent FDA preemption decisions where the Court applied that doctrine in a manner that demonstrates that it should invalidate most state bans or restrictions on mifepristone.

One reason commentators and practitioners alike have difficulty applying the Justices’ opinions on FDA preemption is that their four decisions reflect a somewhat surprising alignment in terms of their usual views on conflicts between federal and state authority. Time and again, the more liberal Justices appointed by Democratic Presidents (Justices Ginsburg, Breyer, Kagan, and Sotomayor) have argued and voted against FDA preemption of state law, even when that state law is being applied in an uncoordinated manner by legislatures and juries.

Conversely—and more importantly given their new ‘majority’ status on the Court—the conservative Justices appointed by Republican Presidents (Chief Justice Roberts, and Justices Alito, Kavanaugh, and Scalia)—in opinions that repeatedly lament ‘uncoordinated’ and ‘parochial’ state regulation—have argued and voted in favor of robust federal preemption of state requirements that conflict with FDA decisions, so as to secure one uniform federal program governing drug approval and use.

And then there is Justice Thomas. He has followed his own unique approach to FDA preemption cases—including one Majority opinion (*Mensing*)—finding FDA preemption where a drug manufacturer could not take any unilateral action that would reconcile competing FDA and state requirements—a likely scenario with most state restrictions on abortion medications.

This article reviews the case for FDA preemption with respect to mifepristone. More so than others who have discussed that issue in the wake of *Dobbs*, in Part I we detail the determinative facts and specific holdings of the four Supreme Court FDA preemption decisions to set forth the principles lower courts must follow—and the Court itself should apply—in the coming litigation over that drug. We especially focus on the pronouncements of the more conservative Justices—most importantly, Justice Alito—given their new ‘majority’ status and because they have generally prevailed in the more recent decisions.

Part II then recounts FDA’s comprehensive program for the balanced, though restricted, use of mifepristone, and the 22 years of Agency actions that brought that about. Unlike prior commentators, we also discuss the details of the recurring interactions between FDA and Congress during the Agency’s long study, approval and regulation of mifepristone—interactions, which evince congressional acceptance of the Agency’s decisions. Given that federal preemption under the Supremacy Clause is highly dependent on Congress’ intent (most often expressed in general authorization legislation, but especially where Congress has reviewed some specific issue), that record will play a critical role in the coming preemption litigation on abortion medications. We then catalog the current state statutes restricting or banning the use of mifepristone, which, in material ways, conflict with that FDA program, which Congress has considered and (at least thus far) left in place.

Part III reviews the competing arguments that will likely be made in future lawsuits seeking preemption of state restrictions on abortion medications—which can range from complete bans to specific requirements that conflict with FDA’s measured actions. We review a recent lawsuit that asserted FDA preemption of Mississippi’s statutes restricting abortion medications—a lawsuit which, although voluntarily dismissed, is a harbinger of the broader litigation to come. We then analyze the strong arguments for FDA preemption of that state’s partial restrictions and then ban of the drug, and address the specific objections raised by the state to application of the Supremacy Clause to those statutes. We conclude that a straight-forward application of the Supreme Court case law—especially the decisions written by Justices Alito and Thomas—should result in FDA preemption of state restrictions, which conflict with the access FDA has mandated.^10^

## I. FDA PREEMPTION DOCTRINE

### I.A. The Comprehensive Nature of FDA Regulation

Few, if any, products are as regulated as pharmaceuticals. Under the Federal Food Drug and Cosmetic Act (FDCA),^11^ FDA has plenary authority over the approval, prescription, and distribution of all drug products.

That regulation begins with FDA’s evaluation of each drug based on years of clinical testing. A company seeking FDA approval must follow a score of detailed regulations to obtain sufficient data from ‘adequate and well-controlled studies’ to establish the safety and efficacy of a proposed drug product,^12^ which is then assembled in a New Drug Application (NDA) running thousands of pages.^13^ The FDA evaluates that data (along with relevant public literature and often prior overseas experience) through a wide range of disciplines including medicine, chemistry, statistics, risk assessment and labeling,^14^ and then approves or disapproves the application.^15^

Even when an NDA succeeds, FDA often conditions its approval on undertakings by the manufacturer to conduct additional, post-approval (Phase 4) studies to assess further risks that were known, but not fully quantified in the NDA, or entirely new risks that may be reported after the drug is first used on a wide scale.^16^ And then, for as long as the drug remains on the market, the Agency monitors its manufacture, promotion, distribution, and all adverse events.

FDA must likewise approve every word, punctuation mark, and typeface in the label that accompanies a drug product and thereby governs its use. Often FDA itself crafts the final language. Every approval letter from FDA incorporates that label—and no deviations are permitted. Thereafter, FDA requires manufacturers to modify their labels whenever there is even an ‘association’ between the drug and some new hazard.^17^

Last, but not least, for the entire time a drug remains on the market, FDA may demand additional restrictions on its use to ‘minimize its risks while preserving its benefits’. These can include demands for revised labeling; patient-oriented package inserts; guides for prescription or dispensing; special packaging; limited refills; restrictions on which health care providers can access the drug; and the suspension or termination of any marketing.^18^

The depth and breadth of this regulatory regime make clear that FDA’s control over pharmaceuticals is not a one-time, binary choice between approval or prohibition, but rather requires the Agency to impose nuanced regulation, which is revised on a continual basis to take account of new data. All of that FDA review and regulation is mandated by a specific organic statute, the FDCA, as interpreted in hundreds of pages of Agency regulations and applied in thousands more of approval decisions, labeling requirements, and risk management plans. That last type of restriction, a ‘Risk Evaluation and Mitigation Strategy’ (REMS),^19^ is a relatively new congressional mandate, added by the Food and Drug Administration Amendments Act of 2007 (‘FDAAA’), which has only been applied to about 60 drugs. But, as discussed below, mifepristone is one of those most closely regulated products.^20^

The REMS amendments to the FDCA direct FDA to work with an NDA-holder to create a set of restrictions on a drug’s use that satisfy six congressionally enumerated factors: ‘The estimated size of the population likely to use the drug’; ‘[t]he seriousness of the disease or condition that is to be treated’; ‘[t]he expected benefit of the drug with respect to such disease or condition’; the ‘expected or actual duration of treatment’; ‘the seriousness of any known or potential adverse events that may be related to the drug’; and ‘whether the drug is a new molecular entity’.^21^

Congress further provided that, in the most difficult cases, FDA can pursue those objectives by also issuing ‘Elements to Assure Safe Use’ (ETASU) restricting use of a drug to providers who have special training, experience or certification; limiting pharmacy dispensation to those who likewise have special certification; limiting use to certain ‘settings’ such as hospitals; and/or mandating enhanced patient monitoring.^22^ At the same time, however, Congress also insisted that such restrictions should not be ‘unduly burdensome on patient access to the drug’ and should ‘to the extent practicable, minimize the burden on the health care delivery system’.^23^

Congress set forth a tight administrative process whereby FDA initially determines that an REMS is appropriate. The NDA-holder must then submit a proposed plan, which FDA reviews against all data on the risks and benefits of the drug. Within 180 days of its receipt of the proposed plan, FDA must issue its REMS as a final agency action.^24^ FDA must thereafter ‘periodically’ reassess each REMS to confirm that it is indeed ensuring patient safety, consistently with the other congressional goals of appropriate access and cost-effectiveness.^25^

Finally, of special importance to future preemption lawsuits, Congress provided that, although either an NDA-holder or FDA can make a proposal to modify an REMS, any such modification requires prior FDA approval. Until such FDA approval, the existing REMS remains in full force and effect.^26^

### I.B. The Statutory Basis for FDA Preemption

Every Supremacy Clause case assessing whether a federal regulatory action preempts some contrary state law or regulation begins with an analysis of the relevant congressional enactment and then the specific administrative actions claimed to have that preemptive effect.^27^ Congress, aware that the comprehensive authority it was conferring on FDA in its 1962 overhaul of the FDCA would raise such preemption issues, chose to address them in a somewhat indirect manner. In Section 202, Congress announced that ‘[n]othing in the amendments [which effectively replaced the 1938 Act] shall be construed as invalidating any provision of State law…unless there is a direct and positive conflict between such amendments and such provision of State law’.^28^

As discussed below, that language, according to the Supreme Court, was intended to preserve traditional ‘conflict’ preemption, under the Supremacy Clause, when it is ‘clear’ that there is a conflict between the mandates of the FDA and some requirement of state law. That view is also supported by the legislative history, which states that Section 202 was intended ‘merely [to] say that this Food and Drug Act shall not be construed as the intent of Congress to abolish all state laws on the same subject where they are not in conflict with the federal law’.^29^ As the Court has also held, assessing the extent of such a conflict—and hence the extent to which some state law requirement may ‘frustrate’ federal objectives—is an intensely factual matter in each case. It is thus essential to review the facts at issue in each of the Court’s four decisions developing and applying the doctrine of FDA preemption.

### I.C. *Levine*

In 2000, Diana Levine was suffering from a severe migraine. She went to a Vermont health center where she was treated by a physician’s assistant operating under the general instructions of an absent doctor.^30^

The assistant administered one dose of the drug Phenergan intramuscularly. When that did not have the desired effect, the assistant administered a second dose directly into Ms Levine’s arm by an ‘IV-push’ of her thumb on a syringe she thought she had placed into a vein. Shortly thereafter, Ms Levine’s arm became gangrenous and was amputated.^31^

Ms Levine’s injury was not a novel one. From the time Phenergan was first approved by FDA in the 1970s, it was known that gangrene can result when the drug is inadvertently injected into an artery. The importance of administering Phenergan only into a vein was therefore set forth in the relevant label. There was likewise language describing a foolproof method of administering the drug intravenously by doing so through an IV-infusion (‘IV-drip’).

In a five-day trial, a Vermont jury heard the story of Ms Levine’s injury; the testimony of the assistant and her supervisor that they would not have used the IV-push method if that method had been formally contraindicated in the Phenergan label; and the dueling opinions of the plaintiff’s expert that the IV-push method should have been so contraindicated versus a defense expert who defended the decision to allow the faster IV-push alternative.^32^

The jury—after being instructed that, under Vermont law, they were to decide the ‘adequacy’ of the Phenergan warnings—returned a verdict for Ms Levine. Wyeth, the manufacturer, then argued that Ms Levine’s claim (that the Phenergan label was deficient in permitting the IV-push method) was preempted by FDA’s approval of the label, which allowed that route of administration. The trial judge and Vermont Supreme Court, however, held that (i) FDA’s review of the administration issue did not show that the Agency would have rejected a label contraindicating the IV-push method; (ii) such a change could have been made by Wyeth unilaterally under FDA’s regulations; and (iii) it therefore could not be said that it was ‘impossible’ for Wyeth to have made that change necessary to satisfy Vermont law within the confines of FDA’s prior decisions.^33^

In 2009, the Supreme Court agreed that Wyeth had failed to make its case for preemption. The Majority Opinion set forth a number of propositions that are still cited today by those opposing FDA preemption in other contexts. But, while *Levine* is the initial, high-water mark of arguments against FDA preemption, the vitality of those propositions has since been diminished (and, some Justices say, overruled) in the Court’s later preemption decisions.

The Justices signing on to the Majority Opinion written by Justice Stevens (joined by Justices Kennedy, Souter, Ginsburg, and Breyer) began by re-stating the question before them: ‘[W]hether the FDA’s drug labeling judgments [as to Phenergan] “preempt state law product liability claims premised on the theory that different labeling judgments were necessary to make drugs reasonably safe for use.”’^34^ The Majority then summarized FDA’s review of the Phenergan label, noting that FDA had approved a version which warned of inadvertent arterial injection and had suggested—but not mandated—the safer infusion method.^35^ Of importance for future lawsuits where a state restriction effectively prohibits some use of a drug, the Majority reasoned that the jury’s verdict did not necessarily require a contraindication of the IV-push method because there may have been ‘any number of ways…to strengthen the Phenergan warning without completely eliminating IV-push administration’, and the Court therefore ‘need not decide whether a state rule proscribing intravenous administration would be pre-empted’.^36^

The Majority then reviewed the manner in which FDA and Wyeth had arrived at the Phenergan label in effect when the drug was administered to Ms Levine. They recounted how Wyeth and the Agency had, in the Court’s view, only ‘intermittently’ corresponded about the label over a 17-year period.^37^ The Majority felt that such ‘passing attention’ by FDA was not sufficient to preempt a Vermont tort law requirement that a different label should have been used under either of the two preemption theories Wyeth had advanced—(i) that it was ‘impossible’ for Wyeth to have complied with both the state and federal law, or (ii) that the state law demand for a different label was an ‘obstacle’ to FDA’s implementation of the ‘purposes and objectives’ expressed by Congress in the FDCA.

The Majority dealt first with Wyeth’s contention that FDA’s approval of the Phenergan label made it ‘impossible’ for Wyeth to issue any alternative warning. There the five Justices relied heavily on an FDA regulation concerning ‘Changes Being Effected’ (CBE). That regulation allowed Wyeth to change portions of the Phenergan label at will—and without prior FDA approval—if the changes were ‘to add or strengthen an instruction about administration…that is intended to increase the safe use of the drug’—language, which clearly covered Ms Levine’s claim that Vermont law required contraindication of the IV-push method.^38^

The Majority Justices were not concerned that the same FDA regulation also provides that ‘the FDA retains authority to reject labeling changes made pursuant to the CBE regulation in its review of the manufacturer’s supplemental application, just as it retains such authority in reviewing all supplemental applications’.^39^ On the facts presented, the Majority did not find ‘clear evidence’ that FDA would have rejected such a change had it been attempted. Posing a hypothetical test that has bedeviled the Court and practitioners ever since, the Majority ruled that ‘absent clear evidence that the FDA would not have approved a change to Phenergan’s label, we will not conclude that it was impossible for Wyeth to comply with both federal and state requirements’.^40^ The Court then summed up its holding on the ‘impossibility’ prong of ‘conflict’ preemption: ‘On the record before us, Wyeth has failed to demonstrate that it was impossible for it to comply with both federal and state requirements. The CBE regulation permitted Wyeth to unilaterally strengthen its warning, and the mere fact that the FDA approved Phenergan’s label does not establish that it would have prohibited such a change’.^41^

The Majority then turned to Wyeth’s alternative argument that ‘recognition of Levine’s state tort action creates an unacceptable “obstacle to the accomplishment and execution of the full purposes and objectives of Congress” because it substitutes a lay jury’s decision about drug labeling for the expert judgment of the FDA’.^42^ The Majority decided, again on the facts presented, that ‘this is not such a case’.^43^

The *Levine* Majority was persuaded by comments from prior FDA Commissioners that FDA’s label requirements did not set a ‘ceiling’ on what a manufacturer can say in a label, but rather only a ‘floor’ which could be supplemented by the manufacturer, for example through a CBE. The Majority likewise cited portions of the legislative history of the FDCA, which, they felt, demonstrated that Congress had left recoveries for drug-induced injuries to tort lawsuits under state law decided by state judges and juries.^44^ The Majority concluded that, ‘Wyeth has not persuaded us that failure-to-warn claims like Levine’s obstruct the federal regulation of drug labeling’.^45^ But the Court then added an important caveat for future cases: ‘[W]e recognize that some state-law claims might well frustrate the achievement of congressional objectives’.^46^

Justice Breyer wrote a separate concurrence to emphasize the Majority’s cautionary note that ‘we have no occasion in this case to consider the pre-emptive effect of a specific agency regulation bearing the force of law’.^47^ He allowed that there might well be future cases where state law ‘interfere[s] with the FDA’s desire’ to impose labeling requirements that ‘serve as a ceiling as well as a floor’, and that, in those circumstances, FDA’s action ‘would have pre-emptive effect’.^48^

In sum, the *Levine* Majority held that, on the facts presented in that particular case, FDA’s review of the Phenergan label did not ‘clearly’ demonstrate that the Agency would have rejected a stronger warning sufficient to satisfy state law. But other cases, based on other facts, might present a different answer to that hypothetical question and hence a different result on preemption.

Justice Thomas, agreeing only with the judgment of the Court, wrote a concurrence making clear that his approach to FDA preemption of state law is quite different. Where the Majority had speculated on whether FDA might have approved a different label had Wyeth submitted one, Justice Thomas focused solely on whether such a change was legally possible.^49^ He too relied on the option of a CBE label change in concluding that Wyeth could have sold Phenergan with the additional warnings required by Vermont tort law, and hence that Ms Levine’s claim was not preempted.^50^

By contrast, the three other conservative Justices endorsed a much more expansive view of FDA preemption. Justice Alito, writing in dissent for Chief Justice Roberts and Justice Scalia, set forth a number of arguments that have since been cited by preemption proponents—and will undoubtedly be used in future lawsuits over state restrictions on abortion medications.

Where the Majority claimed the Court was dealing with only a ‘narrow’ question of whether FDA’s prior approval of the Phenergan label preempted Vermont law requiring a ‘stronger’ label, Justice Alito warned that any warrant to state courts to second-guess FDA on whether a drug is ‘safe as labelled’ undermines the federal system of pharmaceutical regulation: ‘The FDA has long known about the risks associated with IV push in general and its use to administer Phenergan in particular. Whether wisely or not, the FDA has concluded…that the drug is “safe” and “effective” when used in accordance with its FDA-mandated labeling’.^51^

Justice Alito then argued for broad federal preemption. He began by reminding all concerned that the FDA approval process results in a warning label that serves ‘as the standard under which the FDA determines whether a product is safe and effective’ and is thus ‘the centerpiece of risk management’ in all use of the drug.^52^ To Justice Alito and the other conservative Justices, the proper outcome was clear: ‘Where the FDA determines, in accordance with its statutory mandate, that a drug is on balance “safe,” our conflict preemption cases prohibit any State from countermanding that determination’.^53^ The dissenting Justices noted that even the Majority had agreed that the lack of an express preemption provision in the drug portions of the FDCA was ‘irrelevant’ because ‘the ordinary principles of conflict pre-emption turn solely on whether a State has upset the regulatory balance struck by the federal agency’.^54^ According to Justices Alito, Roberts, and Scalia, Section 202 of the FDCA ‘recognizes the background principles of conflict preemption’ and thus ‘would not displace our conflict preemption analysis’.^55^

The dissenting Justices likewise rejected any ‘presumption against preemption’, arguing, on the basis of case law dating back to *Gibbons v Ogden* (1824), that ‘the sole question is whether there is an “actual conflict” between state and federal law; if so, then preemption follows automatically by operation of the Supremacy Clause’.^56^ They observed that the Supreme Court had never held that there was any presumption against preemption ‘in an area—such as drug labeling—that has long been “reserved for federal regulation.”’^57^

The dissenting Justices also disagreed with the Majority on Wyeth’s alternative ‘obstacle’ argument; and they warned that allowing juries, following state law, to second-guess FDA’s labeling decisions would again undermine uniform federal regulation and deprive patients of the benefits of many drugs:

By their very nature, juries are ill equipped to perform the FDA’s cost–benefit-balancing function .…[J]uries tend to focus on the risk of a particular product’s design or warning label that arguably contributed to a particular plaintiff’s injury, not the overall benefits of that design or label… In contrast, the FDA has the benefit of the long view. Its drug-approval determinations consider the interests of all potential users of a drug, including ‘those who would suffer without new medical [products]’ if juries in all 50 States were free to contradict the FDA’s expert determinations.^58^

To document the problems inherent in allowing state law to contradict FDA actions, Justice Alito dove deeply into the record of FDA’s review of the potential risks in permitting the IV-push method to be used under any circumstances. He discussed the recommendations of an FDA advisory committee in 1976 (24 years prior to the use of the method with Ms Levine), which had decided not to contraindicate. He similarly reported on FDA’s subsequent review of the issue in 1987 (13 years before Ms Levine’s injury) and provided cites to the specific articles FDA had considered when it left the Phenergan label with no contraindication.^59^ According to Justice Alito, this administrative record showed enough consideration of the issue by FDA to preempt any state ‘second-guessing’—even though, as the Majority had stressed, there was no ‘clear evidence’ FDA would have rejected such a change had it been suggested by Wyeth at any time over the subsequent decade.

Justice Alito then summed up the dissenters’ core concern: ‘FDA conveys its warnings with one voice rather than whipsawing the medical community with 50 (or more) potentially conflicting ones. After today’s ruling, however, parochialism may prevail’.^60^

*Levine* thus reflects the views of three factions on the Court: The liberal Justices who presume FDA regulation does not preempt state law requirements unless, on the specific factual record, it is ‘clear’ those requirements cannot be reconciled (eg by a label change a manufacturer can make on its own authority). Justice Thomas who decides preemption cases based solely on whether there was some ‘legal’ way for a manufacturer to resolve such a conflict. And the other conservative Justices who much more readily find preemption where there is an apparent conflict between FDA and state law, because those Justices favor one, uniform program of drug regulation by FDA, and FDA alone.

### I.D. *Mensing*

The split between the liberal and conservative Justices in *Levine* proved such an unsure foundation for future FDA preemption decisions that the Court returned three times over the next 10 years to reconcile their views—as the overall balance of the Court shifted toward a more conservative, and here pro-preemption, approach. The first of those three decisions came in 2011 in two companion cases generally cited as *Mensing*.^61^

Both cases involved metoclopramide. While useful in the treatment of gastrointestinal disorders, metoclopramide—like a number of FDA-approved drugs—can, in rare instances, produce irreversible tardive dyskinesia, where patients lose control of certain muscle groups, very often in their face.^62^

The original manufacturer, Wyeth, which first developed metoclopramide and continued to market it under the brand name Reglan, had conferred with FDA from time to time to include warnings about tardive dyskinesia in the Reglan label. Ultimately, in 2009, the risk was elevated to a ‘black box’ at the beginning of the label, which is generally viewed as the strongest form of any warning.

The state law claims in *Mensing* rested on the fact that those changes were not made until after the two plaintiffs had taken the generic version of the drug and had been afflicted with tardive dyskinesia.^63^ When the two cases came respectively before the Fifth and Eighth Circuits, the defendants argued that the plaintiffs’ claims were preempted because, under FDA regulations, they, as generic manufacturers, had no ability to change that label in any way. Both circuits, however, rejected that contention.^64^

Before the Supreme Court, the generic firms pressed their preemption defense, relying on various provisions of the FDCA—including the ‘Hatch-Waxman Amendments’ which had streamlined the approval of generic equivalents to a simple showing that the generic pill was chemically identical to the branded product. The defendant manufacturers stressed that FDA’s regulation of generic drugs rests on the fundamental assumption that a chemical ‘equivalent’ will confer the same benefits, and lead to the same adverse events, as the brand-name version—and that that equivalence is strictly enforced by FDA regulations requiring generic labels to copy the brand-name label in every respect. The generic manufacturers argued that it was thus ‘impossible for them to simultaneously comply with both that federal law and any state tort law duty that required them to use a different label’.^65^ In an amicus brief, the FDA, however, took the position that the generic firms could make such label changes by first approaching FDA with some new safety data and then waiting until the Agency agreed, and was also able to persuade the brand-name competitor that the change was appropriate.^66^

The Supreme Court, in a 5–4 decision—this time with the conservative Justices prevailing—accepted FDA’s interpretation of its regulations. But the Majority nevertheless agreed with the generic manufacturers that plaintiffs’ state law claims were preempted.

The Majority Opinion in *Mensing* was written by Justice Thomas. He and the other conservative Justices (Chief Justice Roberts, Justices Kennedy, Scalia and Alito) believed that the path to a change of a generic label outlined by FDA was too attenuated—and too dependent on other parties—to constitute the type of ‘possible label change’ that was available, through a CBE, to the brand-name manufacturer in *Levine*. Noting that the *Levine* Majority had used the modifier ‘unilateral’ in describing Wyeth’s ‘legal’ ability to make a CBE change, the *Mensing* Majority held that ‘the question for “impossibility” is whether the private party could independently do under federal law what state law requires of it’.^67^ And they therefore rejected the argument that ‘impossibility’ preemption can be defeated by speculating that a manufacturer might escape some conflict between a state law requirement and some FDA action by convincing other parties to go along with some change:

We can often imagine that a third party or the Federal Government *might* do something that makes it lawful for a private party to accomplish under federal law what state law requires of it. In these cases, it is certainly possible that, had the [generic] Manufacturers asked the FDA for help, they might have eventually been able to strengthen their warning label. …If these conjectures suffice to prevent federal and state law from conflicting for Supremacy Clause purposes, it is unclear when, outside of express pre-emption, the Supremacy Clause would have any force. We do not read the Supremacy Clause to permit an approach to pre-emption that renders conflict pre-emption all but meaningless. The Supremacy Clause, on its face, makes federal Law ‘the supreme Law of the Land’ even absent an express statement by Congress.^68^

Conceding that ‘whether a private party can act sufficiently independently under federal law to do what state law requires may sometimes be difficult to determine’,^69^ Justice Thomas immediately added, ‘this is not such a case. …To decide these cases, it is enough to hold that when a party cannot satisfy its state duties without the Federal Government’s special permission and assistance, which is dependent on the exercise of judgment by a federal agency, that party cannot independently satisfy those state duties for pre-emption purposes’—and hence the conflicting state law requirement is preempted.^70^

The *Mensing* Majority then claimed *Levine* was ‘not to the contrary’ because there the Court had relied on the fact that a brand-name manufacturer can use the CBE regulation to change its label independently of anyone or anything else.^71^ The Majority recognized that, from the perspective of a plaintiff who had taken a generic equivalent, rather than the brand-name drug, ‘finding preemption here but not in [*Levine*] makes little sense’—but then declared, ‘it is not this Court’s task to decide whether the statutory scheme established by Congress is unusual or even bizarre’.^72^

All of this infuriated the four liberal members of the Court. Justice Sotomayor, writing for Justices Ginsburg, Breyer, and Kagan, accused the Majority of ‘invent[ing] new principles of pre-emption law out of thin air to justify its dilution of the impossibility standard’, and, in the process, ‘effectively rewrit[ing] our decision’ in *Levine*.^73^

The dissenters expressed their concern that, as the Majority had acknowledged, ‘[a]s a result of today’s decision, whether a consumer harmed by inadequate warnings can obtain relief turns solely on the happenstance of whether her pharmacist filled her prescription with a brand-name or generic drug’.^74^ With a touch of sarcasm, the dissenters said that, ‘The Court gets one thing right: This outcome “makes little sense.”’^75^

The dissenters thus rejected the Majority’s decision to have federal preemption turn on the fact that a brand-name manufacturer can make a label change without prior FDA approval, whereas a generic firm must secure FDA approval before making the same change on the same scientific record.^76^ But, of course, the conservative Justices prevailed in *Mensing*; and that dichotomy is still the law today.^77^

In sum, *Mensing* sets forth a bright-line test (in keeping with Justice Thomas’ approach to preemption): If a manufacturer caught between some FDA decision and a contrary state law requirement can resolve that conflict by ‘unilaterally’ making some change (as a brand-name manufacturer can change its label through a CBE), that may not constitute an ‘impossible’ situation and, under *Levine,* that type of preemption may not lie. But if the manufacturer is not legally entitled to make such a change, independently of FDA or other parties, then ‘impossibility’ preemption will result.

### I.E. *Bartlett*

Two years after *Mensing*, the Supreme Court was again called upon to apply the preemption doctrine in another case involving a generic drug, where the lower courts had denied preemption by reasoning that there can never be an irreconcilable conflict between a state requirement and FDA approval because a manufacturer always has the option of simply exiting the market. Once again, the Supreme Court split along liberal and conservative lines, with the conservatives again carrying the day in an opinion that has been, and certainly will be, used by preemption proponents in future cases, including those involving abortion medications.

The facts in *Bartlett* were, as the Court recognized, ‘horrific’. The plaintiff, seeking relief from a sore shoulder, was prescribed a non-steroidal anti-inflammatory drug, sulindac. Soon thereafter, she developed a rare, but recognized, reaction to the drug, Stevens-Johnson’s Syndrome. Approximately 30 per cent of her skin was ‘burned off or turned into an open wound;’ and she was left in a state of ‘near blindness’.^78^

Ms Bartlett’s lawsuit was founded on the New Hampshire law of ‘design defect’ which, like the law of many states, holds a manufacturer liable for harm resulting from a product if its risks significantly outweigh its benefits, thereby making it ‘unreasonably dangerous’. In *Bartlett*, the jury—reflecting their assessment of a drug that can cause burns and blindness as the price for pain relief—found that sulindac was indeed ‘unreasonably dangerous’ and awarded Ms Bartlett $21 Million.^79^

In its motion for a new trial, the defendant generic manufacturer argued that the state design defect standard was preempted by FDA’s approval of sulindac’s chemical composition and labeling. The district judge, in an opinion issued two months before *Mensing*, disagreed and ruled that Ms Bartlett’s claim was not preempted.^80^ The district judge reasoned that any ‘conflict’ between FDA’s approval of the design of sulindac and New Hampshire ‘design defect’ law could be resolved by simply recognizing that ‘Federal law did not require [the manufacturer] to sell sulindac’ at all.^81^

The First Circuit affirmed, endorsing the same ‘stop selling’ rationale.^82^ The court of appeals conceded that the Supreme Court’s intervening decision in *Mensing* put the defendant manufacturer in a stronger position than it had been when the issue was considered by the district court, but decided that the effect of *Mensing* on the New Hampshire design defect law was best left to the Supreme Court.^83^

In 2013, another 5–4 Supreme Court rejected the ‘stop selling’ rationale and found Ms Bartlett’s use of New Hampshire design defect law was indeed preempted. Writing for all of the Republican-appointed Justices (Chief Justice Roberts and Justices Thomas, Scalia and Kennedy), Justice Alito announced that *Mensing* and other ‘conflict’ preemption decisions, dating back to *McCullouch v Maryland*, established that the New Hampshire state law claim that sulindac was a ‘defective design’ was preempted by FDA’s approval of that very design: ‘[U]nder the Supremacy Clause, from which our preemption doctrine is derived, any state law, however clearly within a State’s acknowledged power, which interferes with or is contrary to federal law, must yield’.^84^

The *Bartlett* Majority assumed that the jury’s finding that sulindac was ‘unreasonably dangerous’ was based on some combination of three factors set forth in the New Hampshire law—‘the usefulness and desirability of the product to the public as a whole, whether the risk of danger could have been reduced without significantly affecting either the product’s effectiveness or manufacturing cost, and the presence and efficacy of a warning to avoid an unreasonable risk of harm from hidden dangers or from foreseeable uses’.^85^ The Majority noted that each of those factors had already been considered by FDA in its approval of sulindac’s chemical composition and label.^86^ And they held that, since the generic manufacturer could not deviate from either the chemical composition or labeling as approved by FDA, the contrary state design defect law was necessarily preempted.^87^

The Majority then flatly rejected the ‘stop selling rationale’ as ‘incompatible with [the Court’s] preemption jurisprudence’.^88^ According to the Majority, all of the Court’s preemption decisions—including *Mensing*—‘presume that an actor seeking to satisfy both his federal- and state-law obligations is not required to cease acting altogether in order to avoid liability. Indeed, if the option of ceasing to act defeated the claim of impossibility, impossibility pre-emption would be “all but meaningless.”’^89^ To Justice Alito and his conservative colleagues, the correct application of the Supremacy Clause and FDA preemption was clear: FDA’s approval of a drug was supreme as against a state restriction or outright prohibition on the sale of that drug. And that preemption could not be avoided by arguing that the manufacturer could simply cease selling a drug that FDA had approved.

In two separate dissents, the four liberal Justices argued that, given the right record, a state law that effectively required a drug manufacturer to choose between enduring damage awards or ceasing its sales of an FDA-approved drug might not be preempted. Justice Breyer, joined by Justice Kagan, reasoned that since a manufacturer could stop selling its product, it was not ‘literally impossible’ for the manufacturer to comply with state law penalizing such sales whenever a jury finds the product was ‘unreasonably dangerous’.^90^ But Justice Breyer also cautioned that a state law yielding ‘sizable damages’ might ‘“stan[d] as an obstacle to the accomplishment of” the federal law’s objective, in which case the relevant state law is preempted’.^91^

Justice Sotomayor, joined by Justice Ginsburg, took a more aggressive line. They rejected the very notion that state tort law conflicts with federal drug law: ‘When a manufacturer cannot change the label or when doing so would not make the drug safe, the manufacturer may still choose between exiting the market or continuing to sell while knowing it may have to pay compensation to consumers injured by its product’.^92^

The *Bartlett* Majority, of course, did not agree. And, in the Majority opinion, Justice Alito responded to the dissenters by arguing that state law damage actions, founded on some evaluation of a drug that is at odds with FDA approval, are effectively the same as statutory prohibitions insofar as they too might contradict FDA approvals—and that both are preempted under the Supremacy Clause—the ‘stop selling rationale’ being no rationale at all.^93^

### I.F. *Albrecht*

*Merck Sharpe & Dohme v Albrecht*,^94^ the last of the Court’s quartet of drug preemption decisions, addressed the open issues of (i) whether impossibility preemption should be determined by a judge or jury and (ii) how ‘clear’ such ‘impossibility’ needs to be. *Albrecht* involved the claims of 500 plaintiffs who had taken Fosamax, a drug used to treat osteoporosis. In a classic example of the ‘good news/bad news’ seen with many pharmaceutical products, the same mechanism of action by which Fosamax inhibits the natural breakdown of older bone cells can, in rare instances, cause otherwise inconsequential, naturally-occurring ‘stress fractures’ to become serious ‘atypical femoral fractures’.

When FDA approved Fosamax, its label did not warn of such serious fractures. But once the drug was prescribed to millions of patients, Merck began to receive reports of such injuries.

In 2008, Merck asked FDA for its approval to add language to the Fosamax label referencing ‘low-energy femoral shaft fractures’. Merck also proposed to cross-reference a longer discussion in another section of the label dealing with the far less serious ‘stress fractures’.

FDA approved the first change, but rejected the proposed cross-reference because the Agency felt the language was ‘inadequate’. FDA invited Merck to submit a revised application addressing the Agency’s concerns. Two years later (2010), the label was ultimately revised to include a more detailed warning about the more serious ‘atypical femoral fractures’—after FDA had ordered that change based on its own analysis of the data.^95^

The 500 plaintiffs who filed individual tort claims under the laws of various states had all taken Fosamax, and suffered serious fractures, between 1999, when the issue was first presented to FDA, and 2011, when the ‘atypical fracture’ language was ultimately added to the label. Merck responded that those state law claims were preempted as a result of the ‘back-and-forth’ between the company and FDA over how best to change the label.

After some preliminary skirmishing, the judge presiding over the 500 cases granted Merck’s preemption motion and dismissed all of the lawsuits.^96^ But the Third Circuit reversed. The court of appeals held that whether the facts of a given case present an instance of ‘impossibility’ is ‘a question of fact for the jury rather than for a judge’. ^97^ The Third Circuit added that, in its view, *Levine’s* requirement for ‘clear evidence’ that FDA would have rejected some label change sufficient to satisfy state law was intended to announce a higher than normal level of proof.^98^

When the case came before the Supreme Court, all nine Justices agreed that whether some action or policy of FDA had preemptive effect is a ‘legal issue’ to be decided by a judge, not a jury. But when it came to articulating the precise question the trial court judges were to decide—and the extent of the ‘evidence’ they were to consider—the Justices disagreed (albeit less strenuously than in their prior decisions.)

The Majority Opinion—written by Justice Breyer, for Justices Kagan, Sotomayor, Ginsburg and Gorsuch—began by ‘describing’ *Levine*.^99^ They stressed that the record there had not shown that Wyeth had kept FDA ‘fully informed’ of the accumulation of adverse event reports and that, as a consequence, FDA had not made an ‘affirmative decision to preserve’ the warnings on the IV-push method or ‘to prohibit Wyeth from strengthening’ them.^100^

Turning to the issue of just how ‘clear’ the evidence must be on the question of ‘impossibility’, the five Justices refused to define the standard any further because, in their view, phrases such as ‘clear and convincing’ might be useful in instructing lay jurors, but not judges who have more latitude and experience in deciding mixed questions of law and fact. They said only that a ‘judge must simply ask himself or herself whether the relevant federal and state laws “irreconcilably conflict”’.^101^ The five Justices added that, in making those determinations, a judge should consider only certain types of ‘evidence’ of FDA ‘action’ where the Agency had announced its conclusions in some relatively formal manner—such as notice-and-comment rulemaking, a rejection of a proposed warning, or ‘other agency action carrying the force of law’.^102^

Having provided that much direction for the lower courts, the Majority declined to decide the specific preemption issue presented. They simply vacated the Third Circuit’s ‘pro-jury’ opinion and remanded the 500 cases for ‘further proceedings’.^103^

By contrast, the four conservative Justices felt the preemption issue in *Albrecht* was easy to decide—although they then split on the proper outcome. For his part, Justice Thomas re-asserted the ‘legalistic’ position he had announced in his concurrence in *Levine*, and then had articulated further in his Majority Opinion in *Mensing*. In his view, Merck had failed to identify ‘any federal law that prohibited [it] from adding any and all warnings…that would satisfy state law’.^104^ He felt that when FDA rejected portions of the initial label change Merck had proposed, and had criticized the company’s focus on mere ‘stress fractures’, the Agency’s letter ‘neither marked “the consummation of the agency’s decision-making process” nor determined Merck’s “rights or obligations.”’^105^

The other conservative Justices—Justice Alito writing for Chief Justice Roberts and Justice Kavanaugh—came out 180 degrees away from Justice Thomas and in favor of preemption. They began their opinion by citing FDA’s own duty to revise warnings whenever the Agency learns of some risk; and they reasoned that, ‘if the FDA declines to require a label change despite having received and considered information regarding a new risk, the logical conclusion is that the FDA determined the label change was unjustified’.^106^ To those Justices, FDA silence following its review of some problem was thus not merely ‘Golden’ but preemptive as well.

In keeping with his abiding preference for FDA preemption so as to produce a single standard for drug manufacturers to follow in all 50 states, Justice Alito then made his own review of the record, marshalling every possible type of ‘evidence’ of Agency ‘action’, including an internal *Merck* memorandum recounting comments apparently made by FDA officials about the optimal label change; email from FDA staff suggesting further negotiations; and a general safety announcement about the fracture risk with the entire class of drugs.

After the case was remanded to the district court, a new judge reviewed the same record and, relying on the same evidence that Justice Alito had marshalled, concluded that Merck had indeed established ‘impossibility’ preemption.^107^ And, in the 3 years since *Albrecht*, the lower courts have followed the general statements of the Majority—and the specific approach of Justice Alito—and have held state law requirements to be preempted whenever the facts show that a ‘fully informed’ FDA approved the use of a drug, which, in some way, conflicts with the state law requirements, and either (i) the manufacturer was not in a position to escape that conflict by undertaking some ‘unilateral’ action it was legally entitled to take, or (ii) there was ‘clear evidence’ FDA would have rejected such a change.^108^ Although it thus took the Court four attempts over 10 years, that is now the standard judges will apply in future preemption cases—including the nascent litigation over state bans or restrictions on abortion medications.

## II. FDA ACTION AND STATE RESTRICTIONS ON ABORTION MEDICATIONS

### II.A. FDA Regulation on Mifepristone

The extent to which the FDA has governed the use of mifepristone far exceeds the degree to which it had regulated the drugs at issue in any of the Supreme Court’s four preemption decisions. That review began in the late 1980s when FDA initially banned the importation of the drug (then known as RU-486) into the United States. That decision generated significant controversy in Congress, and there were a number of hearings challenging it.^109^

The Agency’s position changed under President Clinton; and, in the 1990s, FDA began to consider a New Drug Application for mifepristone. In 1996, FDA submitted the existing clinical data on mifepristone to its Reproductive Health Drugs Advisory Committee—a panel composed of outside medical experts—and asked them to address its safety and efficacy. The advisory committee reviewed the materials and held a public hearing to consider competing points of view.

In addition to the many doctors and public health officials who supported approval, the committee heard from opponents such as the office of Congressman Tom Coburn (R-OK) (a practicing obstetrician), who argued against approval because of the deficiencies he perceived in the clinical data and because the drug ‘takes the life of an innocent unborn baby;’ a spokesperson for the American Life League who stated that ‘[p]regnancy is not a disease and a baby is not a tumor;’ an attorney representing women ‘injured and killed by abortion’, who expressed concerns over how the drug would be prescribed and administered; and Dr Donna Harrison, who challenged the quality and relevance of the clinical data, concluding with a plea to the committee ‘to prevent American women from being used as guinea pigs to satisfy a particular political agenda’.^110^

At the conclusion of the hearing, the advisory committee voted on questions submitted by the FDA staff. The members voted 6 to 0 (with 2 abstentions) that the data then available established the safety and efficacy of mifepristone for use in the United States (although they added they would like to review that data further as more details became available) and that the ‘overall evidence for safety and effectiveness’ of the drug showed that ‘the benefits outweigh the risks for use of the regimen for the proposed indication [abortion] in the United States’. The committee concluded its deliberations by agreeing with the FDA staff that further work should be done to insure the drug would eventually be distributed in a safe manner and that the results of its use would be analyzed in the type of post-marketing studies FDA frequently imposes as a condition of any drug approval.^111^

In response to this favorable initial review by the FDA staff and the Advisory Committee, pro-life members of Congress tried to remove mifepristone from FDA’s authority. In 1998, 1999, and 2000, Rep. Coburn offered amendments to each annual appropriation bill that ‘[n]one of the funds made available in this Act may be used by the Food and Drug Administration for the testing, development, or approval (including approval of production, manufacturing, or distribution) of any drug for the chemical inducement of abortion’.^112^ But while the Coburn amendments passed the House, none was enacted in the final appropriations bills, and FDA was thus allowed to proceed with its review and approval of the drug.

The floor debates on the unsuccessful appropriations prohibitions left no doubt that they were intended to ban abortion medications at the national level. Rep. Coburn and the other opponents of FDA approval made clear their objections were to abortion *per se:* ‘There is something terribly wrong when we ask the taxpayers of this country to spend money in a way which is designed to give the Food and Drug Administration the ability to research and approve drugs that are designed to kill unborn children’.^113^ Other members, who opposed the appropriations riders, such as Nancy Pelosi, were just as clear that their objections stemmed from their desire to make abortion as accessible as possible.^114^

Following the failure of the Coburn amendments, FDA approved the mifepristone (Mifeprex) NDA in September 2000.^115^ The Agency then crafted a label to be used by all prescribers. That initial label stated that the drug could be used in the first 7 weeks of a pregnancy,^116^ and contained a number of contraindications on its use in certain patients for various medical reasons.^117^

In some respects, the Mifeprex label was comparable to most FDA-approved labels, including the ones that have been held to constitute sufficient FDA ‘action’ to preempt state regulation. But the Mifeprex label then went further to include additional restrictions on the drug’s use.^118^ For example, the original label mandated three separate office visits by each patient; that the drug be administered in a ‘clinic, medical office, or hospital by, or under the supervision of, a physician, able to…diagnose ectopic pregnancies;’ that those ‘physicians must also be able to provide surgical intervention in cases of incomplete abortion or severe bleeding, or have made plans to provide such care through others, and be able to assure patient access to medical facilities equipped to provide blood transfusions and resuscitation, if necessary’; and for a follow-up visit 14 days after the drug was used.^119^ All of those steps were substantially more than FDA requires with most other drugs.

Despite these restrictions, FDA’s approval of mifepristone was challenged by anti-abortion groups, most notably the American Association of Pro-Life Obstetricians and Gynecologists (‘AAPLOG’). In August 2002, they filed a Citizen Petition asking FDA to suspend further distribution of the drug until the Agency addressed a number of alleged defects in the approval process, including the supposed inadequacy of the clinical study data; the fact that FDA had followed a particular approval process under Subpart H of its regulations; AAPLOG’s objection to FDA’s decision that mifepristone could be safely used without a prior ultrasound; and the alleged failure of the drug’s manufacturer to comply with the restrictions FDA had mandated.^120^

FDA did not immediately respond to the AAPLOG Petition, but instead worked to refine its restrictions on the distribution of mifepristone. That, in turn, prompted the anti-abortion groups to seek further congressional review of all of FDA’s decisions on mifepristone.

In 2006 a Subcommittee of the House Committee on Government Reform chaired by Rep. Mark Souder (R-Indiana) held a hearing at which pro-life critics and FDA officials debated about the first years of widespread use of Mifeprex.^121^ The Subcommittee again heard from Dr Harrison representing AAPLOG, including her re-submission of the organization’s Citizen Petition.^122^ It also heard from Professor Carter Snead of the University of Notre Dame Law School, who outlined a number of ways FDA could withdraw its approval of mifepristone should the Agency conclude that the various restrictions it had imposed were not sufficient to render it ‘safe’ within the meaning of the FDCA.^123^ But, of significance for the coming litigation over FDA preemption as to mifepristone, neither Professor Snead, Chairman Souder, nor anyone else who spoke at the hearing claimed that FDA did not have the authority to approve the drug or to regulate it like all others. Indeed, the central theme of the pro-life critics in 2006 was that FDA needed to follow those medical and legal principles more closely in regulating mifepristone.

A year later, FDA’s program governing the use of mifepristone was discussed again, this time during the Senate debate on the portion of the 2007 Amendments to the FDCA, which created the REMS program for drugs deemed to require special restrictions. Senator Jim DeMint (R-South Carolina) told his colleagues that he supported the new program but wanted to impose a 7-month deadline for FDA to develop such restrictions for mifepristone.^124^ Again, there was no suggestion that any REMS that FDA might develop was beyond the Agency’s authority because the drug was being used for abortions.

Although FDA did not meet Senator DeMint’s deadline, it did make mifepristone one of the first drugs regulated by an REMS.^125^ In March 2008, FDA reviewed the restrictions set forth in the initial Mifeprex label and determined that they were sufficient to constitute an acceptable REMS.^126^ Not satisfied, Senator DeMint and other pro-life members of Congress asked the Government Accountability Office (GAO)—an independent legislative investigatory body under the House of Representatives—to review FDA’s actions from its initial approval of mifepristone in 2000 through the use restrictions the Agency imposed in early 2008. In August 2008, GAO responded with a 43-page report addressing each of Senator DeMint’s concerns, which, in turn, had closely tracked AAPLOG’s Citizen Petition.

With respect to FDA’s initial approval of mifepristone, GAO compared that decision to the process the Agency had followed with nine other drugs under Subpart H, advising Senator DeMint and the other ‘congressional requesters’ that:

The approval process for Mifeprex was consistent with the processes for the other Subpart H restricted drugs…. Common elements of the approval processes included that FDA needed to evaluate potential limitations in key clinical data (Mifeprex and six of the other drugs), did not approve the drugs in the first review cycle (Mifeprex and five others), and imposed similar types of distribution restrictions on Mifeprex and the other drugs, though the specific details of the restrictions varied across the drugs.^127^

GAO further reported that FDA’s approval of mifepristone was supported by the Agency’s 4-year analysis of clinical studies involving more than 4000 women who had taken the drug; other substantial scientific and medical data analyzed by an FDA review team; additional safety data from the use of the drug in other nations; and the commitments FDA had obtained from the sponsor to undertake various post-marketing studies.^128^

GAO likewise found that the restrictions FDA had imposed on the distribution and use of mifepristone were comparable to those the Agency had imposed on the other drugs approved under Subpart H; that FDA had monitored the sponsor’s compliance with those restrictions (and the results in terms of adverse events) through quarterly, monthly, and 15-day ‘alert’ reports; and that the Agency’s review of the data reported to its Adverse Event Reporting System (AERS) also showed the drug was safe and effective, both as originally approved and in the first 6 years of use.^129^

In 2011, FDA issued a further REMS for the drug, which reinforced and refined the restrictions that had been put in place when the drug was first approved.^130^ A few years later, FDA conducted yet another review of the Mifeprex label and REMS. The Agency assembled internal teams to evaluate the safety data that had been compiled through both the REMS program and FDA’s adverse event reporting system. And in 2016 FDA issued a new version of the REMS, supported by 89 single-spaced pages of scientific analyses, which, in turn, were based on more than 100 clinical studies, journal articles, epidemiological reviews and other medical data.^131^

The 2016 Mifeprex label continued to require that a specially-certified health care provider be responsible for each use of Mifeprex, but no longer dictated where the patient should ingest the second misoprostol pill.^132^ FDA told health care providers to discuss that issue with the patient, recognizing that contractions could begin within 2 hours of taking that second pill.^133^

The revised Mifeprex label now stated that it was safe to use the drug through the first 70 days of pregnancy, rather than the prior limit of 49.^134^ Again, that change was based on FDA’s analysis of clinical study results, which, the Agency found, ‘establish the efficacy of medical abortion with mifepristone and misoprostol through 70 days gestation’.^135^

Again, the pro-life members of Congress referred FDA’s actions to the GAO for further investigation. Specifically, the Republican majority on the House subcommittee overseeing FDA ‘questioned whether the revised Mifeprex labeling has safety implications for women who use the drug’.^136^

In March 2018, GAO responded with another report, which reviewed FDA’s decision to allow use of the drug through 70 days and to eliminate the requirement that the misoprostol component be taken at a medical facility. GAO’s investigation included its own review of the clinical data supporting those changes; a discussion of the competing positions and guidelines of various medical groups (including AAPLOG); and its own audit of FDA’s monitoring.^137^

GAO found that the changes FDA had made in the 2016 REMS and corresponding Mifeprex label were supported by ‘numerous studies’, which ‘collectively evaluated over 35,000 women who took the same dose of Mifeprex and misoprostol approved by FDA;’ that the increase in the time limit to 70 days was similarly supported by 19 studies; and that at-home use of misoprostol was validated in 15 studies (one of which was ‘a large literature review of 87 studies that included over 45,000 women’).^138^

GAO likewise reported that FDA had thoroughly reviewed 15 years of adverse event reports, which ‘demonstrated acceptable safety for the proposed changes to the Mifeprex regimen’ made in the 2016 REMS. GAO noted that those adverse event reports contained 20 deaths among the estimated 3.2 million women who had taken the mifepristone/misoprostol combination—a rate of 0.0006 percent. GAO then added the ‘context’ that ‘a study of mortality among women who did not have an abortion and proceeded to a live birth estimated a mortality rate of 0.009 percent’—15 times greater.^139^

In 2019, FDA approved an Abbreviated New Drug Approval (ANDA) application for mifepristone submitted by a generic competitor, GenBioPro.^140^ Like the manufacturers in *Mensing* and *Bartlett*, GenBioPro was required to sell its generic equivalent under the same terms and conditions as Danco, the NDA-holder—including the same label and REMS restrictions.^141^

In March 2019, FDA received yet another Citizen Petition from AAPLOG.^142^ That one requested that FDA (i) restore, and strengthen, elements of the original mifepristone regimen, as originally announced in 2000, and (ii) continue limiting the distribution of mifepristone to clinics, medical officers, and hospitals—all under the in-person supervision of a certified prescriber.^143^ FDA’s review of those requests coincided with the start of the COVID-19 Public Health Emergency during which FDA recognized that certain REMS requirements for many drugs would be difficult to meet given that ‘patients may need to avoid public places and patients suspected of having COVID-19 may be self-isolating and/or subject to quarantine’.^144^

In April 2021, FDA advised that it was exercising ‘enforcement discretion during the COVID-19 PHE with respect to the in-person dispensing requirement of the Mifepristone REMS Program’.^145^ The Agency also announced that it intended to exercise such enforcement discretion with respect to ‘the dispensing of mifepristone through the mail’ or ‘through a mail-order pharmacy when such dispensing is done under the supervision of a certified prescriber’.^146^ FDA specifically decided that, if all the other requirements of the mifepristone REMS program were met, the Agency would not enforce the in-person requirement for at least the rest of the public health emergency.^147^

In 2020–21, FDA undertook yet another full review of the mifepristone REMS based on information submitted during COVID-19; all new FDA adverse event reporting data; the first four REMS assessment reports; and information submitted by pro-life advocacy groups. Following that review, FDA announced, in December 2021, that it had decided that the ‘in-person dispensing requirement is no longer necessary to assure the safe use of mifepristone…through 70 days gestation’.^148^ FDA found that ‘[r]emoving the in-person dispensing requirement will render the REMS less burdensome to health care providers and patients and…will continue to ensure that the benefits of mifepristone for medical abortion outweigh the risks’.^149^ As before, those conclusions by FDA were substantiated in a 50-page analysis citing scores of studies and other scientific data.^150^

FDA’s December 2021 decision also rejected AAPLOG’s requests (i) to return to the original 49-day time limit; (ii) to limit prescription authority to qualified licensed physicians; (iii) to require a total of three different office visits for each use; (iv) to require additional treatment and advice to Rh-negative patients; and (v) to add contraindication language concerning the availability of appropriate emergency medical care.^151^ Specifically responding to the Citizen Petition request that the drug be dispensed only in certain locations, FDA concluded that the ‘mifepristone REMS Program must be modified to remove the requirement that mifepristone be dispensed only in certain healthcare settings, specifically clinics, medical offices, and hospitals, because this requirement is no longer necessary to ensure that the benefits of the drug outweigh the risks’.^152^ The Agency supported that conclusion with a 12-page review of the prior REMS data, post-marketing safety information, and published literature, which had convinced FDA that the drug could be safely dispensed by retail and mail-order pharmacies; other mail-order clinics; couriers; and ‘partner’ organizations such as ‘Women on Web’ which connect potential patients with such telemedicine groups.^153^

On January 3, 2023, FDA promulgated a revised REMS formalizing the decisions the Agency announced in December 2021.^154^ Building on the prior versions, that latest REMS sets forth a number of requirements that each health care provider must meet to be certified to prescribe the drug.^155^

The new REMS then added language authorizing certified pharmacies to dispense mifepristone directly to patients so long as they comply with the REMS requirements including that they deliver the drug within four days of its prescription; dispense it only in the packaging approved by FDA and provided by the manufacturer; report any patient deaths; and train their staff in the other details of the REMS.^156^

Finally, the revised REMS continues to mandate a number of other actions the mifepristone manufacturers must to take to enforce these prescriber and pharmacy requirements.^157^ But now, consistent with FDA’s decision in December 2021, in-person visits to prescribers, which were suspended in March 2020, are eliminated from the REMS.

### II.B. State Restrictions on the Use of Mifepristone

Against these comprehensive, science-based judgments by FDA as to when, where, how and by whom mifepristone should be administered, a number of states have enacted their own contrary restrictions. Prior to *Dobbs*, there was little enforcement of those statutes, presumably because of *Roe*. But now future judges will need to consider the validity of such state restrictions on other grounds, most obviously the law of FDA preemption.

It appears those judges will be busy, and soon enough. In the year leading up to *Dobbs*—and especially since—governors, state health officials, legislators, and candidates for all the above have been proposing and enacting statutes, which restrict the use of ‘abortion-inducing medications’. Keeping track of those statutes requires a database. And anyone surveying those laws should appreciate the landscape is rapidly changing.

That said, the statutes enacted even prior to the premature release of *Dobbs* can be broadly analyzed in terms of how contrary they are to the mandates and broader purposes of FDA’s regulation. According to a July 2022 listing by the Kaiser Family Foundation, 19 states require the physical presence of the prescribing physician when the patient takes the pills.^158^ Such statutes often specify various parameters the physician must assess during an in-person visit, including the gestational age of the fetus, location of the pregnancy to determine whether it may be ectopic, the patient’s blood type (and an offer of Rh therapy).^159^ According to Kaiser, 28 states (including 9 beyond those requiring the physician’s presence at administration), require a prior ultrasound.^160^

In some states, the requirements for a prior physical exam and in-person administration are coupled with a mandatory in-person follow-up exam 7 to 14 days after administration.^161^ Nine states have statutes explicitly banning the use of telemedicine with mifepristone, including bans on the shipment of abortion-inducing drugs through the mail or via courier.^162^

Other states have formulated their own informed consent forms to be used with the drug. The Kentucky statute, for example, requires a patient be provided with ‘the probable gestational age of the unborn child as determined by both patient history and by ultrasound results used to confirm gestational age’; ‘a detailed list of risks’ of the drug as determined by *Kentucky* health officials; information from a state website on assistance in reversing the effects of the drug; and a dozen more items, culminating with the patient’s acknowledgement that she ‘has a private right of action to sue the qualified physician under the laws of the state of Kentucky if the patient feels coerced or misled prior to obtaining an abortion’.^163^

Finally, some states have issued their own evaluations of the scientific record FDA reviewed when it approved mifepristone to support the state’s contrary restrictions on when the drug may be used. For example, where FDA decided in 2016 to increase the period in which the drug might be administered from the original seven-week limit to ten, Texas still maintains the seven-week limit; Indiana sets the limit at eight weeks; and South Dakota permits nine.^164^

## III. APPLICATION OF THE FEDERAL PREEMPTION DOCTRINE TO THE CONFLICTS BETWEEN FDA REGULATION AND STATE RESTRICTIONS ON MIFEPRISTONE

When compared to the factual and regulatory situations presented in each of the Supreme Court FDA preemption decisions—especially the more recent ones in which the conservative Justices have prevailed—the argument for FDA preemption of state restrictions on the use of mifepristone is strong. But, as made clear by a post-*Dobbs* filing by the State of Mississippi in the first lawsuit to raise FDA preemption with respect to abortion medications, there are counter-arguments that require a full response.

### III.A. The Opening Salvo: *GenBioPro v. Dobbs*

Even before *Dobbs* was decided, the impact of FDA preemption on state laws restricting or banning mifepristone was raised in a lawsuit filed by GenBioPro (GBP), the generic manufacturer, seeking to enjoin Mississippi statutes restricting distribution and use of the drug. That initial lawsuit was voluntarily dismissed by GBP after it bogged down in procedural wrangling. But, just as an overture previews the melodies in the symphony to come, that first lawsuit identified a number of the arguments that will undoubtedly be renewed as the litigation of FDA preemption as to abortion medications resumes, and likely wends its way to the Supreme Court. It is thus worth reviewing the principal issues identified in that case.

In its complaint, filed in October 2020, GBP outlined the history of FDA’s approval of mifepristone, and GBP’s follow-on ANDA, as well as the Agency’s numerous decisions on the restrictions that would best balance the drug’s safe use with reasonable access to its benefits.^165^ GBP charged that the requirements of the 2013 Mississippi statute limiting access to abortion medications ‘squarely conflict[] with the FDA’s REMS conditions for mifepristone on all three broad categories of restrictions: prescribers, safe use, and informed consent’.^166^

First, GBP contended that the requirement in the Mississippi statute that mifepristone be prescribed only by physicians who ‘have completed at least 1 year of postgraduate training in a training facility with an approved residency program and an additional year of obstetrics/gynecology residency’ was contrary to the mifepristone REMS, which authorizes other types of healthcare providers to prescribe the drug. Second, GBP challenged the Mississippi requirements for in-person visits, which ‘effectively ban[] the remote provision of healthcare (often referred to as “tele-medicine”)’ and are therefore contrary to FDA’s express endorsement of that practice. Finally, GBP challenged the part of the 2013 statute creating a special state informed consent form that goes well beyond the form authored by FDA in the mifepristone REMS.^167^

In November 2020, the State responded to GBP’s complaint with a motion to dismiss. The bulk of the motion was a full-throated argument against the merits of GBP’s preemption claim. The State acknowledged that FDA had addressed the same ‘safety’ issues in the mifepristone REMS, but contended that there was still room for the Mississippi legislature to do more.^168^

Although the federal district court heard argument on Mississippi’s dismissal motion in March 2021, the case was effectively stayed once the Supreme Court granted *certiorari* in *Dobbs*. But, as soon as *Dobbs* was decided, GBP moved for leave to file an amended complaint, which would account for the Supreme Court’s decision and add FDA’s December 2021 actions, which, as discussed above, further eliminated restrictions that are still in Mississippi's 2013 statute.^169^

The State opposed GBP’s motion to amend on the grounds that any amendment (or, for that matter, any further litigation) would be ‘futile’ because the more relevant Mississippi statute is now its new ‘trigger law’ which bans all abortions by any means ‘except…where necessary for the preservation of the mother’s life or where the pregnancy was caused by rape’.^170^ The State argued that, under *Dobbs*, that near total ban on abortions was within the State’s authority and was not preempted by FDA’s regulation of mifepristone. The State made a number of arguments future courts will undoubtedly see again as they deal with abortion restrictions in the post-*Roe* world.

First, Mississippi contended that the new ban of virtually any abortion by any means did not conflict with the specific restrictions and balances developed by FDA in its regulation of mifepristone:

[T]he FDCA declares that the FDA’s purpose is to ‘protect the public health by ensuring that …human drugs are safe and effective’.…The trigger law does not second-guess, dispute, or regulate mifepristone to protect the health and safety of pregnant women. Rather than obstruct any federal law or policy, the trigger law prohibits the primary conduct of performing abortions—the act of purposefully destroying an unborn human life.… A State is entitled to prohibit that primary conduct.^171^

Second, Mississippi argued that GBP’s reliance on the mifepristone REMS would only have merit if it were presumed that the FDCA contains some ‘hidden’ grant of authority permitting FDA to ‘effect fundamental changes in areas of “political and economic significance”’ such as abortion: ‘GBP’s preemption claim boils down to the proposition that Congress granted FDA statutory authority to decide that medication abortion should be legal in all 50 States and the FDA has exercised that authority by approving the sale and use of mifepristone. The assertion is preposterous on its face’.^172^ The State specifically argued that such authority could not be found in the 2007 REMS Amendments to the FDCA requiring FDA to ensure that restrictions on the use of a drug are not ‘unduly burdensome on patient access’.^173^

The State likewise asserted that GBP’s preemption claim was not supported by the Supreme Court jurisprudence. Arguing that its defense against preemption was ‘even stronger’ than in *Levine*, Mississippi added that ‘the FDA has never taken the position that the FDCA preempts state regulation of medication abortion procedures’.^174^ The state did all this to oppose even amendment of the pre-*Dobbs* complaint.^175^

On August 18, 2022, GBP informed the district court that it was voluntarily dismissing its 2020 complaint, which had thus bogged down in the need to update its allegations. On January 25, 2023, GBP filed a new complaint in federal court in West Virginia, reflecting all post-Dobbs developments, including the latest REMS, seeking preemption of that state's statutes banning and/or restricting use of mifepristone.^176^

### III.B. Application of the Preemption Decisions to State Anti-Abortion Statutes

A straight-forward application of the ‘conflict’ preemption doctrines set forth in *Levine, Mensing, Bartlett* and *Albrecht* should preempt state bans or restrictions on the use of mifepristone under the Supremacy Clause. ‘Obstacle’ preemption should nullify such state laws because they frustrate the ‘purpose and objective’ of FDA in implementing Congress’ mandate, in both the FDCA and REMS Amendments, to balance safe use and reasonable access to the drug. And ‘impossibility’ preemption should invalidate such state laws where it will be impossible for the drug’s manufacturers to comply with the contradictory federal and state requirements. Put simply, proponents of FDA preemption can effectively argue that where, as here, FDA has said ‘Yes’ to the sale and use of a drug throughout the nation (and has carefully crafted a regimen, which balances the congressional mandates on safety and access), but a state then says ‘No’, the only way a manufacturer can ‘comply’ with those conflicting laws would be to ‘stop selling’—the outcome which the *Bartlett* Court (per Justice Alito) warned would make federal preemption, and hence the Supremacy Clause, ‘all but meaningless’.

#### III.B.1. Bans on Mifepristone

As noted above, there are currently approximately 20 states that have now banned the use of mifepristone within their borders. Some states prohibit mifepristone by name; others by more broadly banning ‘abortion medications’. All of them present serious preemption issues the courts will soon be required to decide.

Mississippi, for example, defines ‘abortion’ as ‘the use or prescription of any instrument, medicine, drug or any other substance or device to terminate the pregnancy of a woman’.^177^ And the state then bans all abortions ‘except in the case where necessary for the preservation of the mother’s life or where the pregnancy was caused by rape’.^178^ Conviction carries a punishment of 1–10 years in prison.^179^ Mississippi further specifically criminalizes the sale or manufacture of any abortion-inducing medication for use in connection with any unlawful abortion.^180^

Idaho’s ban, which went into effect on August 25, 2022,^181^ similarly prohibits ‘chemical abortions’ effected by ‘abortifacients’, which are defined to include ‘mifepristone, misoprostol and/or other chemical or drug dispensed with the intent of causing an abortion’.^182^ Idaho also makes it a felony to ‘provide’ or ‘supply’ any ‘medicine, drug or substance’ to any woman ‘with the intent to cause’ an abortion.^183^ And it is a misdemeanor even to ‘advertise or display for sale anything specially designed to terminate a pregnancy’ (except to a physician or druggist who, as a result of the state ban, are no longer permitted to prescribe or provide the medication).^184^

If they are enforced according to their terms, the Mississippi and Idaho abortion bans—and those in other states like them—will thus ‘directly’ conflict with FDA’s approval of, and detailed REMS for, mifepristone. They will eliminate patient access to an FDA-approved medication that has been deemed so safe and effective that the Agency has now decided it can be used (including through telehealth means) outside the presence of a doctor. And they will inevitably lead to litigation with other states, which are now gearing up to protect doctors, distributors and manufacturers residing in their state from any sanction, by any other state, for prescribing or sending mifepristone across state lines.^185^ In all of that litigation, the courts will need to consider both prongs of conflict preemption.

##### Obstacle Preemption

The proponents of preemption can argue that state bans on mifepristone circumvent FDA’s comprehensive, science-based program reflecting the Agency’s conclusion that it is a safe and effective drug that should be available to women in all fifty states. Such laws thus impede the accomplishment of the ‘purposes and objectives’ of the FDCA and its REMS provisions.

As detailed above, there can be no doubt that FDA has exercised those congressionally-delegated powers in reaching its conclusions concerning the distribution, prescription and use of mifepristone—and that Congress was well aware of what FDA was doing. For more than 20 years, the Agency has reviewed data submitted not only by the manufacturers of mifepristone, but also by opposing parties, such as anti-abortion physician groups, concerning the safety, efficacy and availability of the drug.^186^ FDA then issued hundreds of pages of Agency decisions, which balanced all relevant safety considerations with appropriate access to the drug—just as the Agency was directed to do by Congress in the FDAAA and the FDCA as a whole. As discussed above, those Amendments put ‘assuring access and minimizing burden’ front and center, directing FDA to ensure that its REMS plan will ‘not be unduly burdensome on patient access to the drug’.^187^ Indeed, Congress—which specifically considered FDA’s approval of mifepristone as an abortion drug when it debated and enacted the FDAAA—required FDA to consider ‘patients who have difficulty accessing health care (such as patients in rural or medically underserved areas)’.^188^ FDA’s decisions on mifepristone all along the way have responded to those congressional mandates (and GAO so advised Congress when various pro-life Members asked).

When state law so impedes federal policy, the courts have found obstacle preemption. *Geier v American Honda Motor Co.,*  ^189^ perhaps the preeminent obstacle preemption case, was viewed by both the Majority and Dissent in *Levine* as controlling precedent. *Geier* held that state law could not mandate airbags in all cars when the Department of Transportation (‘DOT’) had promulgated a rule allowing car manufacturers to use a range of options among passive restraint systems.^190^ DOT had previously rejected an ‘all airbag’ standard and permitted a mix of restraints in order to spur technological development and win over consumers.^191^ The Supreme Court decided that a state law claim that car-makers must install airbags presented an obstacle to that federal policy.^192^ As Justice Alito noted, in an extended discussion of *Geier* in his opinion in *Levine*, although federal law did not prohibit the manufacturer from installing airbags in every car, state law still could not compel airbags consistent with the Supremacy Clause.^193^

The same analysis applies to a state ban on mifepristone. Such a ban directly conflicts with FDA’s determination that the drug should be available to all women pursuant to the REMS, just as an ‘all-airbags’ requirement directly conflicted with DOT’s ‘mix of restraints’ determination in *Geier*.

To be sure, the Majority in *Levine* held that obstacle preemption was not established by the facts of that particular case. They did not believe the Vermont tort law requirement for a stronger warning would frustrate any federal ‘objective’ because they found that neither Congress nor FDA had ever indicated that such a warning was not appropriate. But the Court was careful to add that a complete contraindication under some state law of an FDA-approved drug ‘might well frustrate the achievement of congressional objectives’.^194^ State bans on mifepristone present such a case.

Although litigation over state statutes that prohibit an FDA-approved drug is sparse (perhaps because state legislators knew better than to try), there is one notable decision where a federal court struck down such a ban—even though the state was acting under its police power to protect the public health in a manner few would question, were it not for conflicting FDA approval and regulation.^195^ In 2014, Massachusetts Governor Deval Patrick declared a public health emergency and empowered the state Department of Public Health (‘DPH’) to prohibit the sale of Zohydro ER, an extended-release opioid, which had been approved by FDA for the management of severe pain in patients requiring continuous opioid therapy. Zohydro, however, did not possess an ‘abuse resistant formula’, which meant that individuals could crush the pills and then inhale or inject them. DPH, alarmed that such a drug would exacerbate the state’s growing opioid crisis, issued an emergency ban.

The manufacturer, Zogenix, sought an injunction, arguing that the ban was preempted because FDA, not Governor Patrick or the DPH, had the sole authority to approve new drugs and authorize their distribution in interstate commerce. Federal District Judge Zobel agreed and enjoined the state ban.^196^ She held that the ban obstructed ‘FDA’s congressionally-given charge’ to ‘protect the public health’ and to insure that ‘drugs are safe and effective’.^197^ As she explained,

If the Commonwealth were able to countermand the FDA’s determinations and substitute its own requirements, it would undermine the FDA’s ability to make drugs available to promote and protect the public health. The Commonwealth’s emergency order thus stands in the way of ‘the accomplishment and execution of’ an important federal objective. The Constitution does not allow it to do so.^198^

The same is true as to state bans on mifepristone.

##### Impossibility Preemption

 State bans on mifepristone are also vulnerable under the ‘impossibility’ prong of conflict preemption. As discussed above, the four Supreme Court decisions focused primarily on that equally-important type of conflict preemption, which arises ‘where it is impossible for a private party to comply with both state and federal law’.^199^ That has been the type of ‘conflict’ preemption most often asserted by manufacturers in response to claims based on the design or labeling of their drugs. Although the conflicting state requirement in those cases was the common law of tort rather than some statutory law, the touchstone for preemption is the same—whether it is ‘impossible’ for a private party to comply with the requirements of both the federal and state law.

In *Florida Lime and Avocado Growers,*^200^ one of the pillars of impossibility preemption, the Supreme Court posed the since oft-repeated hypothetical: If federal law forbids selling avocados with more than 7 per cent oil content, but California law forbids selling avocados with less than 8 per cent oil content, avocado sellers are put in an ‘impossible’ position.^201^ They cannot sell their avocados in California.

The same is true with a state ban on mifepristone. FDA has approved the drug’s sale across the United States, but some states now forbid it—indeed purport to criminalize it.^202^ The only ‘way’ for anyone to ‘comply’ would be to ‘stop selling’ the drug in those states. But, again, that ‘solution’ is precisely what Justice Alito and the *Bartlett* Majority rejected as ‘incompatible with our pre-emption jurisprudence’. As they made clear, ‘Our preemption cases presume that an actor seeking to satisfy both his federal- and state-law obligations is not required to cease acting altogether to avoid liability’.^203^ And since *Bartlett*, the lower courts have consistently refused to require a company to ‘stop-selling’ a drug FDA has approved.^204^

As for the essential ‘elements’ of such an ‘impossibility’ argument, *Albrecht* provides the formula: Where FDA has been ‘fully informed’ on an issue, and the Agency takes action in approving or otherwise regulating a drug—but a state then seeks to enforce some contrary requirement, such that a private party cannot comply with both—the state law is preempted pursuant to the Supremacy Clause. Indeed, if a state ban of an FDA-approved drug on the grounds that it is being used precisely as FDA dictated does not present an ‘impossibility’ conflict, it is difficult to imagine what does. To quote Justice Alito, with respect to the far less onerous state regulation at issue in *Levine*, ‘The FDA told Wyeth that Phenergan’s label renders its use ‘safe’. But the State of Vermont…said, “Not so.”’^205^

In short, whether analyzed by the doctrines of ‘obstacle’ preemption, ‘impossibility’ preemption, or both, state bans on the distribution and use of mifepristone should be preempted.

#### III.B.2. Limits on the Timing of Use

As discussed above, a few states have laws prohibiting the use of mifepristone before the end of FDA’s 10 week limit.^206^ Given that FDA was ‘fully-informed’ of the alleged safety concerns of using mifepristone up to 10 weeks, and expressly rejected them in December 2021 as not scientifically grounded, any state law imposing a shorter period is vulnerable under both obstacle and impossibility preemption. Apart from the practical problems a distant company would face in policing those different periods of use, a manufacturer that refuses to provide mifepristone to doctors who might use the drug in weeks 7 through 10, because their state had a shorter limit, would be acting inconsistently with the REMS—an FDA mandate the manufacturer is forbidden either to ignore or to modify unilaterally, just as the generic manufacturers could not modify the FDA-mandated labels in *Mensing* or *Bartlett*.

#### III.B.3. In-Person Provider Visits

As described above, the mifepristone regimen most recently revised by FDA does not require a provider to prescribe the medications in-person; nor does it require a patient to take the drugs in the presence of a medical provider. And, while patients are instructed to follow-up with providers approximately 7–14 days later, the FDA likewise does not require that follow-up evaluation be face-to-face.^207^

After reviewing adverse event data and studies related to the in-person dispensing requirements, FDA determined that eliminating them would not increase safety concerns, but rather would ‘reduce [the] burden on patient access and the health care delivery system’ and ‘ensure the benefits of the product outweigh the risks’.^208^ And in its December 2021 decision, and now its 2023 REMS, FDA made those changes permanent. That formal Agency action was grounded in FDA’s rejection of the *2019 AAPLOG Citizen Petition*, which had sought to reimpose in-person requirements.^209^ In that decision, FDA explained why in-person appointments were unnecessary:

Certified prescribers do not have to be physically present with the patient as long as they have confirmed the patient’s gestation age and intrauterine pregnancy…. Moreover, the evaluation of patients for contraindications to medical abortion does not necessarily require direct physical contact with the certified prescriber and can be done in different types of healthcare settings. A certified prescriber can also review the Patient Agreement Form with the patient, fully explain the risks of the mifepristone treatment regimen, and answer any questions, as in any consent process, without physical proximity.^210^

Despite these detailed and science-based FDA determinations—and now their embodiment in the January 2023 REMS—by one recent count, 19 state statutes require the prescribing doctor to be physically present when the patient takes mifepristone; 28 require an ultrasound (which must be done in-person) prior to prescription. And in six states those requirements are coupled with a mandatory in-person follow-up exam 7–14 days after administration.^211^

Such state requirements effectively prohibit telemedicine and the remote healthcare, which FDA, in its REMS, has endorsed for mifepristone. And those state demands ‘directly’ conflict with the Agency’s decisions in 2016, 2021, and now 2023 to expand, rather than restrict, access by suspending in-person requirements. Such state restrictions are thus a legal (and physical) ‘obstacle’ that frustrate FDA’s express goal of access without in-person administration.

They also create an ‘impossible’ situation for manufacturers and distributors who might be held criminally liable for either directly violating, or ‘aiding and abetting’ in violations of, state abortion laws if they provide the drug through FDA’s sanctioned telehealth channels. Yet, as noted above, under the express terms of the FDAAA, neither Danco, the original manufacturer, nor GenBioPro as a generic, is allowed to amend the mandatory terms of the REMS.^212^

The mifepristone manufacturers are thus ‘damned if they do’ send the drug into states in violation of a state restriction, which FDA has expressly rejected, and ‘damned if they don’t’ provide the drug consistently with FDA’s decision to maximize access. The manufacturers will thus be in the same type ‘impossible’ situation that Justice Thomas (and the rest of the Majority in *Mensing*) and then Justice Alito (and the rest of the Majority in *Bartlett*) held to be preemptive.

#### III.B.4. Limiting Prescriptions to Qualified Physicians

After reviewing all of the relevant data, FDA determined in the 2016 REMS that mifepristone was safe and effective when prescribed by mid-level providers (such as physician assistants and nurse-practitioners).^213^ The Agency therefore replaced the term ‘physician’ with ‘healthcare provider’ in the approved labeling.^214^ Five years later, in 2021, FDA rejected AAPLOG’s petition seeking to reimpose the ‘physician-only’ requirement. Once again, FDA concluded the ‘available information establishes that mid-level healthcare providers as well as physicians can determine whether mifepristone is an appropriate treatment for a particular patient and dispense it’.^215^

Despite these findings, 29 states bar anyone other than physicians from prescribing mifepristone.^216^ Some statutes go even further and limit prescribers to physicians who are obstetricians/gynecologists,^217^ those who have training in surgical abortion,^218^ and/or those who have admitting privileges at a hospital within a short distance from their office.^219^ Such restrictions on who can prescribe mifepristone will effectively prohibit its use in many areas—particularly rural ones—where no physician meets the requirements. The statutes thus impose obstacles to FDA’s fulfillment of Congress’ mandate in the FDAAA to maximize patient access consistent with safe use. And such statutes once again put manufacturers in the ‘impossible’ position of either following FDA’s decision that such special training is not necessary or refusing to provide the drug to such doctors in order to obey state law.

#### III.B.5. Additions to Informed Consents

As noted above, since the 2011 REMS, FDA has required the use of a ‘Patient Agreement’ as one of the ‘elements to assure safe use’ (ETASU) for mifepristone. That form, created by FDA itself, sets forth instructions for use, information about the risks and potential complications, and steps to take in an emergency.^220^

During FDA’s 2016 REMS review, Danco requested that the Patient Agreement be removed from the ETASUs as unnecessary. The FDA review team agreed, finding that the Patient Agreement is ‘duplicative of information in the Medication Guide and of information and counseling provided to patients under standard informed consent practices for medical care and under professional practice guidelines’.^221^ But the FDA Commissioner overruled that recommendation, and the Patient Agreement was retained.^222^ He decided the form ‘would not interfere with access and would provide additional assurance that the patient is aware of the nature of the procedure, its risks, and the need for appropriate follow-up care’.^223^

This history shows that FDA has generally supported the practice of other organizations providing additional information about mifepristone and seeking informed consent from patients. Assuming those organizations are acting to assure the safe use of the drug—rather that to deter patients from taking it at all—such additional information may be complimentary to, and consistent with, the Agency’s REMS, and thus would not pose an obstacle to FDA’s purpose or conflict with FDA’s existing regimen. In such a case, the defenders of such additional terms may be able to argue that there is no ‘clear evidence’ FDA would reject their addition to FDA’s own form.

That said, some states have imposed more onerous informed consent requirements, which contain elements that arguably conflict with the mifepristone REMS.^224^ Those provisions will need to be reviewed one-by-one to determine whether they contradict FDA’s program for the safe and effective use of mifepristone—including the express congressional purpose of maximizing appropriate access. Again, as the case law makes clear, this is an area where ‘facts matter’, and some additions to informed consents may pass muster, while others may not.

### III.C. The Contention that a State’s General Right to Regulate Abortions Precludes FDA Preemption of Restrictions on Abortion Medications

States seeking to ban or restrict the use of mifepristone and other abortion medications will attempt to avoid any ‘obstacle’ or ‘impossibility’ analysis by broadly arguing that, because the subject is abortion, such laws are exempt from the doctrine of FDA preemption and the Supremacy Clause on which it is based. Mississippi already has.

As noted above, in its opposition to GBP’s preemption complaint, Mississippi argued that its ban on almost any use of mifepristone was only part of its general policy against abortion *per se*, and thus, according to the State, ‘does not conflict with or obstruct [the] purpose’ of FDA in regulating all drugs sold in the United States.^225^ Mississippi argued that, ‘The trigger law does not question FDA’s determination that mifepristone is safe and effective. The trigger law does not second-guess, dispute, or regulate mifepristone to protect the health and safety of pregnant women’. The State thus assumed that preemption arises under the Supremacy Clause only when a state is addressing the same legal issues addressed in some conflicting federal law.

Some authors have commented that the case for preemption may be most difficult where a state, such as Mississippi, has backstopped its restrictions on the use of mifepristone with a general ban on abortions. Professors Cohen, Greer and Rebouche suggest the case for preemption may be ‘uncertain’ in such circumstances.^226^ But, following additional analysis in which they consider the congressional purpose, expressed in the REMS legislation, that drugs requiring special federal restrictions should still be made as available as possible, they appear to conclude that the case for FDA preemption of the distribution of mifepristone is viable.^227^

Professor Zettler has likewise suggested that ‘challenges to all abortion care on the ground that such laws are preempted by the FDCA, at first glance, seem less likely to succeed than challenges brought against drug-specific restrictions or bans’.^228^ But she too ultimately observes—primarily on the basis of Justice Alito’s reasoning in *Bartlett*—that the ‘courts could conclude that, while states retain significant power to regulate the practice of medicine, they do not have the power to regulate medical practice in ways that make compliance with FDA requirements impossible, or obstruct the purposes of the FDCA’.^229^

In our view, there are a number of serious flaws in any argument that a general state ban on abortions supersedes the preemptive effect of FDA’s congressionally-mandated regulation of mifepristone. As a preliminary matter, we note that many states with statutes that restrict the use of mifepristone do not have any such ‘morality’ counter-argument because their legislatures have not banned abortions prior to the 10-week limit FDA has set for the drug’s use. Specifically, 13 states, with about 27 per cent of the nation’s population (Arizona, Florida, Indiana, Kansas, Michigan, Nebraska, North Carolina, North Dakota, Ohio, South Carolina, Utah, Wisconsin and Wyoming), permit abortions for at least as long (and generally longer) than the 10 weeks during which mifepristone may be used. Yet those states nevertheless have statutes that conflict with the regimen set forth in the latest mifepristone REMS. Most require one or more office visits (often with mandatory ultrasounds) before and/or after administration; others specifically prohibit the use of telemedicine in the dispensation of the drug. Those typically pre-*Dobbs* statutes often have legislative findings or history announcing the views of state legislators on the safety of the mifepristone/misoprostol combination that materially conflict with the findings of FDA in its REMS. As shown above, they are thus preempted by FDA’s detailed actions to the contrary.

Then there are 13 other states, with about 22 per cent of the nation’s population (Alabama, Arkansas, Idaho, Kentucky, Louisiana, Mississippi, Missouri, Montana, Oklahoma, South Dakota, Tennessee, Texas and West Virginia), which ban virtually all abortions from conception, thus covering the first 10 weeks in which FDA has authorized the use of mifepristone. (Georgia, with about 4 per cent more of the population, is a ‘middle’ case in that it is theoretically possible mifepristone might be used within the 6 weeks allowed for abortions in that state, but less than the 10 weeks FDA has approved). Beyond the overall bans, most of these states also have specific statutes against the telehealth regimen FDA has now endorsed for the drug. Just as Mississippi did in the early *GBP* lawsuit, those states will presumably argue that the conflict between those more specific statutes and the provisions of FDA’s REMS is now ‘irrelevant’ because of those general bans.

That argument, however, is not supported by either the text of the Constitution or the case law applying it to preempt conflicting state law—including, of course, state laws that conflict with the actions of a ‘fully informed’ FDA. While a state statute that conflicts with, or acts as an obstacle to the accomplishment of, some federal law may most obviously do so when it speaks in the same terms as the federal law, such an identity of goals or authority is not necessary for preemption. The Supremacy Clause contains no exception for a state law enacted for some reason different from the federal law with which it conflicts: ‘The Laws of the United States…shall be the Supreme Law of the Land; and the Judges in Every State Shall be Bound thereby, any Thing in the…Laws of any State to the Contrary notwithstanding’.^230^

That is also clear from the prior Supremacy Clause cases followed by the Supreme Court in its FDA preemption decisions. For example, the *Levine* Majority relied on *Fidelity Federal Savings v de la Cuesta*, a case involving the conflict between federal bank regulations and a provision of California real property law.^231^ In that case, California argued that the federal regulations could not preempt a state statute on a subject so clearly reserved to the states. But the Court, applying the Supremacy Clause, flatly rejected that argument and held that, ‘These principles are not inapplicable here simply because real property law is a matter of special concern to the States: “The relative importance to the State of its own law is not material when there is some conflict with a valid federal law, for the Framers of our Constitution provided that the federal law must prevail.”’^232^

*Fidelity Federal*, in turn, had relied on *Free v Bland*, where the Supreme Court ruled that a provision of Texas community property law was preempted by a federal Treasury regulation regarding saving bond ownership, despite the traditional and exclusive state interest in matrimonial law.^233^  *Free*, a unanimous decision of the Warren Court, summed up 140 years of Supremacy Clause cases, dating back to *Gibbons v Ogden,*^234^ holding that ‘any state law, however clearly within a State’s acknowledged power, which interferes with or is contrary to federal law, must yield’.^235^ As noted above, Justice Alito quoted the same language in his opinion for the Court in *Bartlett* (although he attributed the quotation to the Court’s more recent decision in yet another case on preemption, *Gade v National Solid Waste Management*—a case involving the conflict between certain state standards for licensing hazardous waste site workers and differing standards set by EPA).^236^ In short, as the Court held as recently as *Bartlett* (and in the specific context of FDA preemption), whether the state interest is in regulating real property, matrimonial relations, river boat licenses, the qualifications of hazardous waste workers, or drugs approved by FDA, the fundamental principle of constitutional law remains the same—under the Supremacy Clause, conflicting state law ‘must yield’, and the ‘relative importance to the State of its own law is not material’.

The statutory basis for preemption in the FDCA is likewise not limited to state requirements ostensibly founded on the safety, efficacy, or availability of drug products. As discussed above, Section 202 of the FDCA rests preemption on whether there is a ‘direct and positive conflict’ between the state and federal law. If there is, the state law is preempted, regardless of the basis of the state law.

That too is clear from the four Supreme Court decisions on FDA preemption. In each case, the conflicting state law requirement was one of state tort law. As discussed above, those requirements were typically set forth in common law decisions that did not even mention pharmaceuticals. And no one—surely no member of the Supreme Court—doubted that each state had an interest in maintaining and enforcing its own tort law to compensate its citizens harmed by the negligence of others. By contrast, and as acknowledged in those decisions, federal drug law does not provide any relief for someone injured by an FDA-approved drug. Put simply, the federal government regulates drugs; state law compensates tort victims. Nevertheless, as each of those four decisions recognized, when the mandates of state negligence law are applied in a way that conflicts with the decisions of the FDA with respect to some drug, those state laws, as applied, are preempted, without regard to the fact that tort law has traditionally been left to the states.

### III.D. The Contention that FDA Preemption of State Laws Banning or Restricting FDA-Approved Abortion Medications Presents a ‘Major Decision’ No Court Can Make

As a corollary to its reliance on the fact that, under *Dobbs*, the states have general authority to regulate abortions, Mississippi argued in its *GBP* pleadings that contrary FDA preemption would mean that ‘Congress granted FDA authority to decide that medication abortion should be legal in all 50 states and that FDA has exercised that authority by approving the sale and use of mifepristone’.^237^ To support its assertion that such an interpretation of the FDCA would be ‘preposterous’, Mississippi relied on the Supreme Court’s recent decision in *West Virginia v EPA*,^238^ and argued that such an interpretation of the FDCA would constitute a ‘major decision’ no court can make.

That argument, however, ignores the many critical differences between the situation at issue in *West Virginia* and FDA’s regulation of mifepristone. In *West Virginia*, the Court held that EPA’s novel position that it could use its authority in the Clean Air Act, over the emission of pollutants, to require coal-burning power plants to reduce their production of electricity and to promote alternative forms of generation (wind, solar) was not only contrary to the text of that statute, but also constituted a ‘major decision’ Congress had not left to EPA’s discretion. In reaching that conclusion, the Court stressed that (i) EPA’s novel interpretation was something quite different from anything EPA had ever done before; (ii) EPA claimed to find that ‘hidden’ authority in an ‘ancillary provision’ of the Clean Air Act, which had ‘rarely been used in the preceding decades, and never for that purpose;’ and (iii) EPA was exercising authority that Congress had, in fact, refused to grant EPA on at least four separate occasions.^239^

As detailed above, the 22-year history of FDA’s approval and regulation of mifepristone is markedly different in every respect. FDA’s initial approval of Mifeprex in 2000, and its follow-on approval of GBP’s generic equivalent in 2019, were issued with the full knowledge of all concerned that the medication’s purpose was to terminate pregnancies. FDA made those decisions pursuant to specific provisions of the FDCA and hence its long-standing authority from Congress to do so. Those decisions by FDA applied the central—not ‘ancillary’—provisions of the FDCA, just as the Agency had done as to other drugs hundreds of times before. And on at least three occasions, Congress specifically voted on whether to authorize FDA to ‘spend the taxpayers’ money’ to study and potentially approve mifepristone as an abortion drug; and, each time, Congress voted to do so.

FDA then implemented the REMS provisions in the FDAAA in its 2016 and 2021 decisions, creating a comprehensive program for the distribution, prescription and administration of mifepristone—a program supported by medical science and balancing factors expressly mandated by Congress. As discussed above, many of the specific issues addressed in those FDA decisions had been openly debated as far back as the congressional hearings in 2006. But no one at those hearings contended FDA was not authorized by Congress to apply the standards and requirements of the FDCA and FDAAA to mifepristone. Indeed, during the debate on the passage of the FDAAA in 2007, Senator DeMint, a fierce opponent of abortions, stated that he was supporting that legislation, and specifically its REMS provision, because he wanted FDA to use that authority to regulate the drug. And after the Agency did so, he asked GAO to investigate and advise Congress on whether FDA was doing so properly. And GAO did.

There is thus no way to claim Congress never addressed FDA's authority to use its traditional powers over all drugs to approve and regulate an abortion drug, or that Congress did not know how the Agency was exercising that authority. On the contrary, Congress was well aware that FDA was reviewing, approving, and regulating mifepristone under its general authority over all drugs; Congress took votes on that issue; and those votes authorized FDA to proceed.

The same fact-based analysis would apply if a state rests such a ‘major decision’ objection on the Supreme Court’s rejection of FDA’s attempt to assert authority over tobacco products in *Brown & Williamson* (the principal precedent cited by the Court in *West Virginia*).^240^ There the Court—in considering Commissioner Kessler’s novel claim that FDA already had authority to regulate cigarettes as ‘drug delivery devices’—focused on 90 years of legislative and administrative history, which established that Congress in fact never intended to confer such authority on the Agency.

Again, a comparison of the two situations demonstrates that *Brown & Williamson* has no relevance to FDA’s regulation of mifepristone. In *Brown & Williamson* the Court began with a 9-page explanation of the many ways the requirements for the approval of a drug under the FDCA would necessarily result in a total ban of cigarettes—a result which, the Court recognized, ‘would contradict Congress’ clear intent’ expressed in many other statutes to regulate, but not remove them from the market.^241^ The Court then spent 15 more pages recounting the many times Congress had refused to accede to requests from health officials to extend the FDCA to include tobacco products,^242^ at times relying on ‘FDA’s consistent and repeated statements that it lacked authority under the FDCA to regulate tobacco…’.^243^ None of those facts so critical in *Brown & Williamson* is present in FDA’s long regulation of mifepristone under the FDCA. Again in this case Congress plainly authorized FDA to approve and regulate mifepristone.

Nor is there anything in the Court’s decision in *Dobbs* to overrule *Roe* that prevents the courts from enforcing the Supremacy Clause (which, of course, was not at issue in *Dobbs*) and ruling that FDA’s actions regulating mifepristone preempt state statutes restricting, or banning, its use. As the opinions of both the Majority and Justice Kavanaugh (who provided the critical fifth vote to overrule *Roe*) repeatedly stress, the stated goal of *Dobbs* was to insure that abortion policy will be made by democratically-elected officials, rather than judges and Justices: ‘After today’s decision the nine Members of this Court will no longer decide the basic legality of pre-viability abortion for all 330 million Americans. That issue will be resolved by the people and their representatives in the democratic process in the States or Congress’.^244^ FDA’s authority over mifepristone is undeniably the product of such democratic processes. The FDCA and REMS Amendments were enacted by majority votes of Congress, which, of course, is composed of members elected by the voters. And the FDA Commissioners who applied those federal statutes likewise served at the pleasure of a democratically-elected President. Should the people (voters) be dissatisfied with any FDA action as to mifepristone, they can elect a President who will replace those officials or elect new members of Congress who can change the FDCA or FDAAA.

Indeed, even prior to *Dobbs*, the prospect of federal regulation of medicated abortions, above and beyond the FDA regulation to date, was raised in bills submitted by both sides to the abortion debate. In 2021, pro-life members of Congress proposed the Teleabortion Prevention Act, which would fine or imprison healthcare providers who provided a ‘chemical abortion’ without ‘physically examining the patient;’ ‘being present at the location of the chemical abortion;’ or without scheduling a follow-up visit for the patient.^245^ The proposed act—which failed to clear the House Judiciary Committee—thus would have effectively prohibited providing the drugs by telemedicine.

On the other hand, pro-choice members of Congress introduced the Women’s Health Protection Act—which passed in the House in September 2021, but failed to secure cloture on a 46 to 48 vote in the Senate in February 2022. That bill would have backstopped the preemptive effect of FDA approval and regulation by specifically nullifying any state law limiting a provider’s ability to prescribe or dispense abortion medications based on ‘current evidence-based regimens;’ limiting abortion services via telemedicine ‘other than a limitation generally applicable to the provision of medical services via telemedicine;’ or requiring that a patient make ‘one or more medically unnecessary in-person visits to the provider’.^246^ Although those two bills were not enacted, Congress is still free to pass other legislation. And that is all *Dobbs* requires.^247^

## IV. CONCLUSION

Although neither a complete nor perfect solution, a definitive ruling that FDA regulation of abortion medications preempts more restrictive state statutes would meet a number of the objections set forth in *Dobbs*, while insuring meaningful access to those drugs.

First, by invoking the Supremacy Clause, as the Supreme Court has done in other cases to preempt state laws that conflict with FDA regulation, such a ruling would rest on an unassailable constitutional foundation—the type of foundation the *Dobbs* Majority said was missing in *Roe*. The protection of such FDA preemption would be easiest to invoke in states that continue to permit abortions in limited circumstances—ie up to and, in many cases, well beyond FDA’s 10 week limit on the use of mifepristone—but still have restrictions (eg on telehealth) which FDA has specifically rejected.

The enforcement of the Supremacy Clause will be more profound in states with statutes that ban abortion entirely. There, of course, the stakes are even higher, including the decidedly uncivil war that may well break out if one state tries to sanction health care providers in another state for sending an FDA-approved drug across state borders. A single, preemptive federal policy on abortion medications (with the limits also imposed by FDA) could thus offer a constitutionally based resolution of such otherwise explosive ‘state versus state’ disputes.

Second, the use of mifepristone protected by FDA preemption would, by virtue of FDA’s own restrictions, be limited to the first 10 weeks of gestation. To many that might present a more acceptable situation than the later-term surgical procedures, which the *Dobbs* Majority—and Chief Justice Roberts in his concurrence—felt were most troubling to pro-life advocates.^248^ Those now engaged in increasingly bitter debate in a post-*Roe* nation might find some room to compromise in that more moderate environment.

Finally, as just noted, a ruling recognizing FDA preemption of state laws unduly restricting or outright banning abortion medications would not be vulnerable to the constant refrain in *Dobbs* that *Roe* was ‘undemocratic’ because it ‘wrongly removed an issue from the people and the democratic process’.^249^ Instead the courts would be doing nothing more—nor less—than faithfully following the Supremacy Clause to protect decisions made by FDA, under the FDCA as enacted by Congress, from the state ‘parochialism’ decried by Justice Alito and the other conservative Justices.

To be sure, that last aspect of FDA preemption also reveals its less than permanent nature. Future FDA Commissioners (or the Presidents at whose pleasure they serve) could decide for ‘democratic’ (that is, political) reasons to change FDA’s policy towards abortion medications (although the terms of the FDCA and the requirements of the Administrative Procedures Act could check truly arbitrary decisions). Indeed, if the electorate so demanded, Congress could change the FDCA to exclude abortion medications from FDA’s authority (as some members unsuccessfully tried to do before mifepristone was approved); or Congress might come to some different policy on FDA regulation of abortion medications (as Congress did after *Brown & Williamson* by creating a new set of standards for regulating tobacco.) But, in that event, the courts could still faithfully follow the Supremacy Clause by enforcing that new policy Congress ‘democratically’ mandated, rather than—as Justice Alito warned in *Levine*—allowing the ‘parochialism’ of uncoordinated state statutes to usurp federal authority over which drugs may be used throughout the nation.

## CONFLICT OF INTEREST

As noted in the initial notation, after this article was originally published on SSRN, Ms O’Connor was retained to assist GenBioPro, Inc. in various litigation including *GenBioPro v. Sorsaia*, Case No. 2:23-cv-11111 (S.D.W.V. Jan. 25, 2023).

## Footnotes

1
*Dobbs v Jackson Women’s Health Organization*, 142 S. Ct. 2228 (2022).

2 ‘This Constitution, and the Laws of the United States which shall be made in Pursuance thereof…shall be the Supreme Law of the Land; and the Judges in every State shall be bound thereby, any Thing in the Constitution or Laws of any State to the Contrary notwithstanding.’ U.S. Const. Art. VI, cl. 2.

3 In 2020, medication abortions accounted for 54% of all abortions, up from 39% in 2017. Guttmacher Institute, *Medication Abortion Now Accounts for More than Half of All US Abortions* (Feb. 2022).

4 Department of Justice, *Attorney General Merrick B. Garland Statement on Supreme Court Ruling in Dobbs v Jackson Women’s Health Organization* (June 24, 2022).

5 The explosion of telehealth distribution of mifepristone began even before the premature disclosure of Justice Alito’s Majority opinion. As discussed below, over the last two years, FDA has increasingly authorized the mailing, and in-home use, of mifepristone, encouraging services such as Aid Access, Hey Jane, and Abortion on Demand to send the drug across state lines. *See* NPR, *Telehealth Abortion Demand is Soaring. But Access May Come Down to Where You Live* (May 20, 2022)

6 Clarke D. Forsyth and Donna Harrison M.D., *State Regulation of Chemical Abortion After Dobbs,*  Liberty University Law Review, 16:377, 391–404 (June 2022).

7
*See*  *id*. at 405–10.

8 555 U.S. 555 (2009) discussed, *infra* at 7–11.

9 Forsyth, *State Regulation*, *supra*, at 411–13.

10 Since *Dobbs* was decided, three articles (in addition to the Liberty Law Review paper discussed above) have considered federal preemption of state statutes banning or restricting abortion medications. In August, Professors Cohen, Donley and Rebouche revised a prior draft of their omnibus article surveying the many issues raised by *Dobbs* to address the prospect of such FDA preemption. David S. Cohen, Greer Donley, Rebecca Rebouche, *The New Abortion Battleground*, 123 Columbia Law Rev. (forthcoming) (Aug. 30, 2022), https://ssrn.com/abstract=4032931 at 43–59. They generally share our view that state statutes restricting the use of mifepristone should be preempted. As discussed below, those authors do express some uncertainty about the strength of the preemption argument in the 13 states that now ban abortions entirely; but they ultimately seem to agree preemption is viable even in those circumstances. *Id.* at 53–54. (As a related matter, those authors also believe some of FDA’s remaining restrictions on the dispensation of mifepristone should be eliminated. See *id*. at 75–77. *See also* Greer Donley, *Medication Abortion Exceptionalism*, 107 Cornell Law Review 627, 651 (2022). We, by contrast, assume that those FDA restrictions will remain in force, and that those arguing in favor of FDA preemption of conflicting state requirements will therefore be in an even stronger position, as preemption generally turns on the extent to which a federal agency has imposed decisive regulation. In September, Professor Lewis Grossman briefly reviewed the relevant case law in a ‘Weekend Briefing’ in the New England Journal of Medicine, concluding that the extent of FDA preemption of such state restrictions is an ‘open’ legal issue. Lewis Grossman, *Pushing Back with Pills—Enhancing Access to Reproductive Health Drugs After **Dobbs*, NEJM (Sept. 17, 2022). In November—the same week a draft of this article was first posted on SSRN (https://ssrn.com/abstract=4258890)—Professor Patricia Zettler and colleagues posted a draft of their analysis of potential FDA preemption of state restrictions on mifepristone. Patricia Zettler et al*.*, *Mifepristone, Preemption, and Public Health Federalism* (forthcoming in the Journal of Law and the Biosciences), https://ssrn.com/abstract=4267498. *See also* Patricia Zettler and Ameet Sarpatwari, *Perspective: State Restrictions on Mifepristone Access – The Case for Federal Preemption,* 386 NEJM 705 (Feb. 24, 2022). Although Professor Zettler and her colleagues do not detail the controlling Supreme Court decisions, nor recount the congressional acquiescence as to FDA’s many actions on mifepristone, they also ultimately agree that the proper outcome of such future litigation *should* be federal preemption of conflicting state statutes. *Id*. at 25–37.

11 Drug Amendments of 1962, Pub. L. No. 87–781, 76 Stat. 780 (1962).

12 FDA, *Guidance for Industry: Providing Clinical Evidence of Effectiveness for Human Drug and Biological Products*, (May 1998) https://www.fda.gov/media/71655/download.

13 Pub. L. No. 75–717, §§ 505(d)-505(e), 52 Stat. 1040, 1052–53 (1938).

14 FDA, *New Drug Application (NDA)*, (Jan. 21, 2022) https://www.fda.gov/drugs/types-applications/new-drug-application-nda.

15 21 C.F.R. § 314.110 (2021).

16 21 U.S.C. § 355(o) (2021).

17 21 C.F.R. § 201.57(e) (2021).

18 Congressional Research Service, *FDA Risk Evaluation and Mitigation Strategies (REMS): Description and Effect on Generic Drug Development* (Mar. 16, 2018) at 5–8. Significantly, the mifepristone REMS was one of just two selected by CRS as prime examples to highlight for Congress. *Id*. at 7.

19 21 U.S.C. § 355–1.

20 Mifepristone terminates a pregnancy by preventing attachment to the uterine wall. Misoprostol, another drug regulated by FDA, taken 24–48 hours later, prompts contractions which expel the uterine contents.

21 21 U.S.C. § 355–1(a)(1)(A-F).

22 21 U.S.C. § 355–1(f)(3).

23 21 U.S.C. § 355–1(f)(5)(A)(ii) & (B)(ii). See also *id*. at § 355-1(g)(2)(C)(ii).

24 21 U.S.C. § 355–1(a)(2)(B), (f)(5), (h)(1).

25 21 U.S.C. § 355–1(f)(5).

26 21 U.S.C, § 355–1 (g)(1), (h)(1), (h)(2)(A), (B).

27
*See*  *Wyeth v Levine*, 555 U.S. 555, 565 (2009).

28 Drug Amendments of 1962, Pub. L. No. 87–781, 76 Stat. 781, 793 (1962).

29 108 Cong Rec 21,083 (1962)(remarks of Rep. Howard Smith).

30
*Levine v American Home Products, Inc.,* 2004 WL 5456809 (Vt. Super. July 30, 2004).

31
*Levine v Wyeth*, 183 Vt. 76, 82 (Vt. 2006).

32
*Levine v American Home Products, supra*, 2004 WL 5456809 at 2–3.

33
*Id.* at 3–7; 183 Vt. 76, 82–99.

34
*Wyeth v Levine*, 555 U.S. 555, 563 (2009).

35
*Id.* at 560, n. 1.

36
*Id.* at 565.

37
*Id*. at 561–62.

38
*Id.* at 568.

39
*Id*. at 581.

40
*Id*. at 571.

41
*Id.* at 573.

42
*Id*. at 563–64 *(quoting Hines v Davidowitz,* 312 U.S. 52, 67 (1941)).

43
*Id*. at 581.

44
*Id*. at 577–79.

45
*Id*. at 580.

46
*Id*. at 581.

47
*Id.* at 582.

48
*Id.*

49
*Id.* at 582–83.

50
*Id.* at 592–94.

51
*Id*. at 606.

52
*Id.* at 608 (*quoting* 50 Fed. Reg. 7470 (1985)).

53
*Id*. at 609.

54
*Id.*

55
*Id.* at 612, n. 4.

56
*Id.* 623–24 and n. 14.

57
*Id*. at 624, n. 14.

58
*Id.* at 626 (*quoting Reigel v Medtronic*, 552 U.S. at 326).

59
*Id*. at 613–17.

60
*Id.*

61
*PLIVA, Inc. v Mensing*, 564 U.S. 604 (2011).

62
*Id*. at 609–10.

63
*Id*. at 610.

64
*Mensing v Wyeth, Inc.*, 588 F.3d 603, 614 (8th Cir. 2009); *Demahy v Actaavis, Inc.*, 593 F.3d 428, 449 (5th Cir. 2010).

65 564 U.S. at 610.

66
*Id*. at 617.

67
*Id*. at 620 (*citing Wyeth*, *supra*, 555 U.S. at 573).

68
*Id*. at 620–21.

69
*Id*. at 623.

70
*Id*. at 623–24.

71
*Id*. at 624.

72
*Id*. at 625 (*quoting Cuomo v Clearing House Assn, L.L.C,* 557 U.S. 519, 556 (2009) (Thomas, J. concurring in part and dissenting in part)).

73
*Id*. at 627.

74
*Id*.

75
*Id*.

76
*Id*. at 637.

77 In response to *Mensing*, the Obama Administration proposed a new FDA regulation that would ‘create parity’ between brand-name and generic firms by authorizing the generic manufacturers to ‘independently make’ label changes. 78 Fed. Reg. 67,985, 67,988–89 (Nov. 13, 2013). Such a ‘unilateral’ change—without the necessity of *prior* FDA approval—would presumably persuade Justice Thomas that there was no longer any basis for ‘impossibility’ preemption. *Id*. at 67,989–91. FDA’s proposed regulation, however, was opposed by both the brand-name and generic manufacturers. A final rule was never issued; and eventually FDA withdrew the proposal. 83 Fed. Reg. 64,299 (Dec. 14, 2018).

78
*Bartlett v Mutual Benefit Pharmaceutical Company*, *Inc.,* 678 F.3d 30, 34 (1st Cir. 2012).

79
*Bartlett v Mutual Benefit Pharmaceutical Company, Inc.,* 760 F. Supp. 2d. 220 (D.N.H. 2011).

80
*Id*. at 246–50.

81
*Id*. at 248.

82 678 F.3d. 30 (1st Cir. 2012).

83
*Id*. at 38.

84
*Mutual Pharmaceutical v Bartlett*, 570 U.S. 472, 480 (2013) (*quoting Gade v National Solid Wastes Management Assn.*, 505 U. S. 88, 108 (1992)).

85
*Id*. at 483 (*quoting Vautour v Body Masters Sports Industries, Inc*., 174 N. H. 150, 154 (2001)).

86
*Id*. at 483–86.

87
*Id*. at 486–87.

88
*Id.* at 488.

89
*Id.* (*quoting Mensing, supra,* 564 U.S. at 621).

90
*Id*. at 493.

91
*Id*. at 494 (*quoting Crosby v National Foreign Trade Council*, 530 U.S. 363, 373 (2000)).

92
*Id*. at 511.

93
*Id.* at 490–92.

94 139 S. Ct. 1668 (2019).

95
*In re Fosamax*, 951 F. Supp. 2d 695, 697 (D.N.J. 2013).

96
*In re Fosamax*, 2014 WL 1266994 (D.N.J. Mar. 26, 2014).

97
*In re Fosamax*, 852 F.3d 268, 282 (3rd Cir. 2017).

98
*Id*. at 284–85.

99 139 S. Ct. at 1676–78.

100
*Id*. at 1678 (*quoting Levine, supra*, 555 U.S. at 572).

101
*Id*. at 1679 (*quoting Rice v Normal Williamson, Co.,* 458 U.S. 654, 659 (1982)).

102
*Id*. at 1689.

103
*Id*. at 1680–81.

104
*Id*. at 1682.

105
*Id*. at 1683 (*quoting Bennett v Spear*, 520 U.S. 154, 178 (1997)).

106
*Id*. at 1684.

107
*In re Fosamax Products Liability Litigation*, MDL No. 2243, 2022 WL 855853 (D.N.J. Mar. 23, 2022).

108
*See*, *eg*  *Knight v Boehringer Ingelheim Pharms. Inc.*, 984 F.3d 329, 338–41 (4th Cir. 2021) (manufacturer did not have ‘newly acquired information’ to warrant a unilateral label change); *Dolin v GlaxoSmithKline LLC*, 951 F.3d 882, 891 (7th Cir. 2020) (manufacturer disclosed relevant data and FDA ‘unambiguously rejected’ a stronger warning); *Cerveny v Aventis, Inc.*, 855 F.3d 1091, 1099 (10th Cir. 2017) (FDA’s rejection of a citizen petition was ‘clear evidence’ FDA would not have approved the desired warning); *In re Incretin-Based Therapies Prod. Liab. Litig.*, 524 F. Supp. 3d 1007, 1033 (S.D. Cal. 2021) (‘Given the specific attention the agency has given to the very matter at issue in this litigation’, FDA effectively informed manufacturers that it would reject a label change), *aff’d*, No.21–55,342, 2022 WL 898595 (9th Cir. Mar. 28, 2022).

109
*See eg*  *RU 486: The Import Ban and Its Effect on Medical Research*, Hearing Before Subcomm. on Regulation, Business Opportunities and Energy of the Committee on Small Business of the House of Representatives, 101st Cong., 2d Sess. (1990).

110 Food and Drug Administration, Reproductive Health Drugs Advisory Committee, Hearing on New Drug Application for the Use of Mifepristone for Interruption of Early Pregnancy (July 19, 1996), Tr. 145–48, 156–59, 183–86, 246–50.

111 Minutes of the FDA Reproductive Health Drugs Advisory Committee (July 19, 1996) at 5–7.

112 144 Cong. Rec. H5089–5100 (June 24, 1998); 145 Cong. Rec. H3798–3812 (June 8, 1999); 146 Cong. Rec. H5693–5709 (July 10, 2000).

113 145 Cong. Rec. H3798 (June 8, 1999).

114
*See, eg* 144 Cong. Rec. at H5099 (June 24, 1998).

115
*FDA Approval Letter* (Sept. 28, 2000), https://www.accessdata.gov/drugsatfda_docs/appletter/2000/20687appltr.pdf.

116
*2000 Mifeprex Label* (Indications and Usage), https://www.accessdata.fda.gov/drugsatfda_docs/label/2000/20687lbl.pdf.

117
*Id.* (Contraindications).

118 The original label was approved under Subpart H regulations (21 C.F.R. §14.520 (2021)) which allows restrictions on its distribution to assure the drug’s safe use.

119
*2000 Mifeprex Label* (Dosage and Administration).

120
*Citizen Petition re: Request for Stay and Repeal of the Approval of Mifeprex (mifepristone) for the Medical Termination of Intrauterine Pregnancy Through 49 Days’ Gestation,* submitted by American Association of Pro-Life Obstetricians and Gynecologists, the Christian Medical Association and Concerned Women for America (Aug. 2002) (‘*2002 AAPLOG Citizen Petition’*), https://aaplog.org/wp-content/uploads/2021/01/2002-Aug-Citizen-Petition_Mifeprex-8.20.02.pdf.

121
*RU-486: Demonstrating a Low Standard for Women’s Health?*, Hearing Before the Subcommittee on Criminal Justice, Drug Policy, and Human Resources of the Committee on Government Reform, House of Representatives, 109th Cong., 2nd Sess. (May 17, 2006).

122
*Id.* at 138–337. As noted above, Dr Harrison is one of the authors of the recent *Liberty Law Review* article which argues that the same sort of restrictions should now be imposed through state statutes.

123
*Id.* at 338–345.

124 153 Cong. Rec. 5469, 5470 (May 2, 2007).

125 21 U.S.C. § 355–1.

126 73 Fed. Reg. 16,313 (Mar. 27, 2008).

127 U.S. Government Accountability Office, Report to Congressional Requesters, *Food and Drug Administration: Approval and Oversight of the Drug Mifeprex* (GAO-08-751) (Aug. 7, 2008) at ‘Highlights’. *See also*  *id*. at 30–42.

128
*Id*. at 15–25.

129
*Id*. at 25–43.

130
*2011 Mifeprex Risk Evaluation & Mitigation Strategy* (June 8, 2011), https://www.accessdata.fda.gov/drugsatfda_docs/rems/Mifeprex_2011-06-08_Full.pdf.

131
*2016 Mifeprex Medical Review* (Center for Drug Evaluation & Research) (Mar. 29, 2016), https://www.accessdata.fda.gov/drugsatfda_docs/nda/2016/020687Orig1s020MedR.pdf.

132
*2016 Mifeprex Label* (Dosage and Administration), https:/www.accessdata.fda.gov/drugsatfda_docs/nda/2016/020687Orig1s020Lbl.pdf

133
*Id.*  *See also*  *2016 Mifeprex Medical Review* at 6, 38–41.

134
*2016 Mifeprex Label* (Dosage and Administration).

135
*2016 Mifeprex Medical Review* at 5, 20–38.

136 U.S. Government Accountability Office, Report to Congressional Requesters, *Food and Drug Administration: Information on Mifeprex Labeling Changes and Ongoing Monitoring Efforts* (GAO-18-292) (Mar. 28, 2018) at 2.

137
*Id*. at 1–4.

138
*Id*. at 12–14.

139
*Id*. at 17–21.

140
*2019 ANDA Approval Letter for Mifepristone* (Apr. 11, 2019), https://www.accessdata.fda.gov/drugsatfda_docs/appletter/2019/091178Orig1s000ltr.pdf

141
*See*  *Mensing,* 564 U.S. at 612–13 (citing 21 U.S.C. §§ 355(j)(2)(A)(v) & 355 (j)(4)(G)).

142
*Citizen Petition* submitted by AAPLOG and American College of Pediatricians (Mar. 29, 2019) (“*2019 AAPLOG Citizen Petition)*, https://aaplog.org/wp-content/uploads/2021/01/Citizen-Petition-Final-FDA-Mif-REMS.pdf.

143
*Id.* at 1.

144
*See*  *FDA Response to AAPLOG Citizen Petition*, Docket No. FDA-2019-P-1534 (Dec. 16, 2021) at 5.

145
*Letter from Janet Woodcock, M.D.* at 2 (Apr. 12, 2021), https://www.aclu.org/sites/default/files/field_document/fda_acting_commissioner_letter_to_acog_april_12_2021.pdf.

146
*Id.*

147
*Id.*

148
*FDA Response to AAPLOG Citizen Petition* at 6. The World Health Organization, reflecting use of the drug in other nations, has now counseled that mifepristone may be used, without any in-person office visits, for up to 12 weeks. *W.H.O. Abortion Care Guideline,* § 3.6.2, *Self-Management Recommendation* 50 (2022).

149
*FDA Response to AAPLOG Citizen Petition* at 6.

150
*Id.*

151
*Id*. at 9, 13–15, 18–19.

152
*Id*. at 25.

153
*Id*. at 25–36. For one study supporting the safety of telehealth administration of the drug, *see* Ushma Upadhyay et al., *Safety and Efficacy of Telehealth Medication Abortion in the US During the COVID-19 Pandemic*, JAMA Network Open (Aug, 24, 2021), https://jamanetwork.com/journals/jamanetworkopen/fullarticle/2783451.

154
*FDA, Risk Evaluation and Mitigation Strategy (REMS)* (Jan. 3, 2023).

155
*Id*. at 1–2.

156
*Id*. at 3–4.

157
*Id*. at 4.

158 Kaiser Family Foundation, *State Requirements for the Provision of Medication Abortion*, https://www.kff.org/womens-health-policy/state-indicator/state-requirements-for-the-provision-of-medication-abortion/ (last accessed on Aug. 30, 2022). The 19 states that contradict FDA’s conclusion that it is not only not necessary, but indeed can be problematic, to insist that the prescriber be present (because that might mean contractions and expulsion will occur while the patient is travelling home) are Alabama, Arizona, Arkansas, Indiana, Kansas, Kentucky, Louisiana, Mississippi, Missouri, Nebraska, North Carolina, North Dakota, Oklahoma, South Carolina, South Dakota, Tennessee, Texas, West Virginia, and Wisconsin.

159 Texas, Health & Safety Code Ann. § 171.063(c). See also Alabama, Ala. Code § 26-23E-7; Arkansas, Ark. Code Ann. § 20-16-1504(c)(1); Indiana, Ind. Code Ann. § 16–34–2-1; Mississippi, Miss. Code Ann. § 41–41-107(2), (3); Missouri, Mo. Ann. Stat. § 188.021(1); Montana, Mont. Code Ann. § 50–20-707(1); North Dakota, N.D. Cent. Code Ann. § 14–02.1-05.3; South Dakota, S.D. Codified Laws § 36–4-47; Tennessee, Tenn. Code Ann. § 63-6-1104(a); Wisconsin, Wis. Stat. Ann. § 253.105(2).

160 In addition to the 19 states noted above which require the physician to be present at administration, the ultrasound requirement may adversely affect the full use of telemedicine as to mifepristone in Florida, Georgia, Idaho, Iowa, Michigan, New Hampshire, Ohio, Utah, and Wyoming.

161 See Arkansas, Ark. Code Ann. § 20-16-1504(f); Mississippi, Miss. Code Ann. § 41–41-107(5),(6); Montana, Mont. Code Ann. § 50–20-707(3); South Dakota, S.D. Codified Laws § 36–4-47; Tennessee, Tenn. Code Ann. § 63-6-1104(c); Texas, Health & Safety Code Ann. § 171.063(e).

162 Arizona, Ariz. Rev. Stat. Ann. § 36–3604; Arkansas, Ark. Code Ann. § 20-16-1504 (b); Louisiana, La. Stat. Ann. § 40:962.2; Missouri, Mo. Ann. Stat. § 188.027; Montana, Mont. Code Ann. § 50–20-704; South Carolina, S.C. Code Ann. § 40–47-37(c)(6); Tennessee, Tenn. Code Ann. § 63-6-1103; Texas, Health & Safety Code Ann. § 171.063(b-1); West Virginia, W. Va. Code Ann. § 30–1-26.

163 Kentucky, K.R.S. 311.7735(4)(k). *See also* Montana, Mont. Code Ann. 50–20-707; Oklahoma, 63 Okla. Stat. Ann. § 1–756(c).

164 Texas, Health & Safety Code, § 171.063(c)(6); Indiana, Ind. Code Ann. § 16–34–2-1; South Dakota, S.D. Codified Laws § 36–4-47.

165
*GenBioPro, Inc. v Dobbs*, Civ. No. 3:20-CV-652-HTW-LRA (S.D. Miss.), Complaint (Oct. 9, 2020) at 5, 9–18.

166
*Id*. at 18 (*citing* Miss. Code Ann, §§41–41–101-117).

167
*Id*. at 18–23.

168
*GenBioPro*, *supra,* Memorandum of Authorities in Support of Motion to Dismiss (Nov. 6, 2020) at 6–10, 17–20.

169
*GenBioPro*, *supra*, Memorandum in Support of Its Motion for Leave to File Amended Complaint (July 21, 2022).

170
*GenBioPro*, *supra*, Defendant’s Memorandum in Opposition to Plaintiff’s Motion for Leave to File Amended Complaint (Aug. 4, 2022) at 4 (*quoting* Miss. Code Ann. § 41–41-45)(2).

171
*Id. at 8–9 (citing Dobbs, supra).*

172
*Id*. at 12–13.

173
*Id*. at 13.

174
*Id*. at 17.

175 Mississippi also asserted that ‘the fact that Congress has criminalized the shipment of abortion drugs by mail and by common carriers traveling in interstate commerce dooms GBP’s claim that these laws are preempted.’ *Mem. Opp*. at 15–16. The State cited two sections of the Federal Criminal Code (18 U. S. C. §§ 1461, 1462). Those two sections can be traced back to the Comstock Act of 1873 which prohibited the mailing of ‘obscene’ or ‘immoral’ items as well as drugs or other articles `for causing unlawful abortion.' Act of Mar. 3, 1873, ch. 258, 17 Stat, 598. Section 1461 continues to prohibit the mailing of items `intended for producing abortion.' And section 1462 prohibits the use of an express company or other common carrier for transporting such items. But, beginning in the early twentieth century, the federal courts recognized that the statutes did not apply to products or articles used by physicians for lawful purposes. As the Second Circuit explained, those provisions ‘…were part of a continuous scheme to suppress immoral articles and obscene literature and should so far as possible be construed together and consistently. If this be done, the articles here in question ought not to be forfeited when not intended for an immoral purpose.’ *United States v One Package*, 86 F.2d 737, 739 (2nd Cir. 1936)(citing cases in agreement from the Sixth and Seventh Circuits). The Second Circuit held that the provisions were ‘not to prevent the importation, sale, or carriage by mail of things which might intelligently be employed by conscientious and competent physicians for the purpose of saving life or promoting the well-being of their patients.’ *Id*.; *see also Application of the Comstock Act to the Mailing of Prescription Drugs that Can Be Used for Abortions, *46 *O.L.C. Slip Op. *__ at 17 (Dec. 23, 2022) (DOJ Office of Legal Counsel concluding that `section 1461 does not prohibit the mailing of articles that can be used to produce abortion, including mifepristone and misoprostol, where the sender lacks the intent that those items should be used unlawfully' and `recipients in every state can use them lawfully in some circumstances'). Today, of course, even more than in the early 1900s, abortions are often lawful depending on a number of factors, which vary from state to state, including gestational age. Enforcement of these provisions as to mailed abortion drugs—assuming federal officials would wish to do so—will thus depend on the legality of the use of the medication, as determined, *inter alia*, by sound application of FDA preemption under the Supremacy Clause—not the other way around. *See id.* at 3 n.5 (reserving on the further issue of `the possible effect of federal law on' state laws restricting `the mailing of these drugs for abortion purposes within their jurisdiction').

176
*GenBioPro v. Sorsaia, *Case No. 2:23-cv-11111 (S.D.W.V. Jan. 25, 2023).

177 Miss. Code Ann § 41–41-45(1).

178 Miss Code Ann. § 41–41-45(2). The statute only recognizes rape as an exception ‘if a formal charge of rape has been filed with an appropriate law enforcement official.’ Miss. Code Ann. 41–41-45(3).

179 Miss. Code Ann. § 41–41-45(4).

180 Miss Code. Ann. § 97–3-5 (any ‘person who sells…or offers to sell…any drug or medicine, for causing unlawful abortion…or who manufactures any such article or medicine, is guilty of a misdemeanor, and, on conviction, shall be punished by fine…and by imprisonment’).

181 Idaho Code § 18–622. *But see*  *U.S. v Idaho*, Memorandum Decision and Order, Case No. 1:22-cv-00329-BLW (D. Id. Aug. 24, 2022) at 38 (granting preliminary injunction against enforcement of Idaho’s abortion ban to the extent it conflicts with the federal Emergency Medical Treatment and Labor Act).

182 Idaho Code §18–617(1)(a)

183 Idaho Code § 18–605(1).

184 Idaho Code § 18–607.

185
*Dobbs* has already provoked a number of states to enact ‘shield’ laws protecting providers within their borders from actions brought to enforce the anti-abortion prohibitions or restrictions of other states. The Massachusetts law, the most comprehensive to date, proclaims that “[a]ny public act or record of a foreign jurisdiction that prohibits, criminalizes, sanctions, authorizes a person to bring a civil action against or otherwise interferes with a person, provider, carrier or other entity in the commonwealth that seeks, receives, cause, aids in access to, aids or abets or provides…reproductive health services [defined to include ‘the termination of a pregnancy’]…shall be a violation of the public policy of the commonwealth.” H.B. No. 5090 (July 25, 2022), § 4(c). The Massachusetts law provides that anyone subjected to any ‘litigation…to deter, prevent, sanction or punish’ them from assisting in ‘the exercise and enjoyment…by any person of rights to reproductive health services’ can sue for ‘injunctive, monetary, or otherwise appropriate relief’, which can include quashing any process, and obtaining damages — including the amount of any fine levied in the underlying litigation, expenses and attorneys’ fees in connection with such litigation. *Id*. § 4(d). The new law also immunizes health care providers from any medical board discipline, ‘including the revocation, suspension or cancellation of the certificate of registration, or reprimand, censure or monetary fine, for providing’ abortions. *Id*. § 14. Finally, the law provides that Massachusetts courts will not enforce judgments of other states issued ‘in connection with any litigation concerning legally-protected health care activity’, or extradite providers to some opposing state. *Id*. §§ 33–34.

186 See *2002 AAPLOG Citizen Petition; 2019 AAPLOG Citizen Petition*.

187 21 U.S.C. § 355–1(f)(2) (2021).

188 21 U.S.C. § 355–1(f) (2021).

189 529 U.S. 861 (2000).

190
*Id.* at 875.

191
*Id.* at 878–79.

192
*Id.* at 881.

193
*Levine*, 555 U.S. at 611 (Alito, J., dissenting). *See also*  *Fidelity Fed. Sav. & Loan Ass’n v de la Cuesta,* 458 U.S. 141, 155 (1982) (‘The conflict does not evaporate because the [agency’s] regulation simply permits, but does not compel’ the action forbidden by state law).

194
*Levine*, *supra*, 555 U.S. at 583.

195
*Zogenix, Inc. v Patrick*, 2014 WL 1454696 (D. Mass. Apr. 15, 2014).

196
*Id.* at ^*^3.

197
*Id*. at ^*^2.

198
*Id. (citations omitted*).

199
*Crosby v Nat’l Foreign Trade Council,* 530 U.S. 363, 372 (2000).

200
*Florida Lime and Avocado Growers, Inc. v Paul,* 373 U.S. 132 (1963).

201
*Id.* at 1217–18.

202
*See* Idaho Code Ann. § 18–607; Miss. St. Ann. § 97–3-5.

203
*Bartlett*, 570 U.S. at 487 (citing *Mensing, supra*, 564 U.S at 621).

204
*See*  *Utts v Bristol-Myers Squibb Co.*, 251 F. Supp. 3d 644, 678 (S.D.N.Y. 2017) (‘In approving [the drug] for manufacture and distribution, the FDA determined that [the drug] was safe and effective for its indicated use….’ Thus, claims that ‘challenge[] the FDA’s approval of…[an] indication…are preempted’), *aff’d*, 919 F.3d 699 (2d Cir. 2019); *Gross v Pfizer, Inc.*, 825 F. Supp. 2d 654, 659 (D. Md. 2011) (there is ‘no state law duty that would compel generic manufacturers to stop production of a drug that under federal law they have the authority to produce. Nor could such a state law duty exist, as it would directly conflict with the federal statutory scheme in which Congress vested sole authority with the FDA to determine whether a drug may be marketed in interstate commerce’), *aff’d*, 741 F.3d 470 (4th Cir. 2014); *Yates v Ortho-McNeil-Janssen Pharms., Inc.*, 808 F.3d 281, 300 (6th Cir. 2015) (state law claim ‘that defendants should never have sold the[ir] FDA-approved [product] in the first place’ was preempted under *Bartlett* as another variant of a ‘“stop-selling” rationale’); *Silver v Bayer Healthcare Pharms., Inc.*, No. 2:19-CV-3495-DCN-MHC, 2021 WL 4472857, ^*^4 (D.S.C. Sept. 30, 2021) (plaintiff’s claim that the defendant’s drug ‘should have been banned…constitutes a “stop-selling” theory, which courts have consistently found to be preempted by federal law’); *Trisvan v Heyman*, 305 F. Supp. 3d 381, 405 (E.D.N.Y. 2018) (‘“an outright ban” cannot be a viable alternative to sustain a [state-law] claim.’ (internal citation omitted)); *Utts v Bristol-Myers Squibb Co.*, 226 F. Supp. 3d 166, 186 (S.D.N.Y. 2016) (‘Insofar as the plaintiffs’…suggest[] that the defendants should never have sold the FDA-approved formulation of [their drug], such claims have been explicitly repudiated by the Supreme Court.’); *In re Lipitor (Atorvastatin Calcium) Mktg., Sales Pracs. & Prods. Liab. Litig.,* 185 F. Supp. 3d 761, 771 (D.S.C. 2016) (‘any claims that Defendant should have simply stopped selling the drug to women…is preempted’); *Mayor & City Council of Baltimore v GlaxoSmithKline, LLC,* No. 24-C-20-004788, 2022 WL 537004, at ^*^4 (Md. Cir. Ct. Jan. 28, 2022) (municipality ban on entire class of drugs because they were ‘inherently dangerous’ preempted because it was grounded on the contention that the defendants “would violate their duty under [state] law by simply marketing [their drugs] in its FDA-approved form and with its FDA-approved label).

205 The argument above is consistent with Professor Lars Noah’s general discussion of FDA preemption law, prior to the current focus on state statutes restricting abortion medications. In *State Affronts to Federal Primacy in the Licensure of Pharmaceutical Products*, 2016 Mich. State L. Rev. 1, 27–34 (2016), he cites the language quoted above from Justice Alito’s opinion in *Bartlett* to argue that, ‘if the relatively more attenuated command of design defect scrutiny in tort law created an actual conflict with federal law governing FDA-approved drugs, then surely such an outright sales prohibition imposed by state officials would do so.’ *Id*. at 35.

206
* See*  *supra* at 27–28 (South Dakota, Indiana and Texas impose 7, 8 and 9 week cut-offs, respectively).

207
*2016 Mifeprex Label* (Dosage and Administration § 2.3).

208
*2021 FDA Questions and Answers on Mifeprex*, No. 5.

209
*FDA Response to AAPLOG Citizen Petition* at 12–19 (‘the findings show medical abortion at home is both safe and effective without three office visits’).

210
*Id.* at 12.

211
* See*  *supra* at 27.

212
* See*  *supra* at 6; 21 U.S.C. § 355–1(g)(1), (h)(1), (h)(2)(A) & (B).

213
*2016 REMS Medical Review* at 7.

214
*Id.*

215
*FDA Response to AAPLOG Citizen Petition* at 11.

216 Kaiser Family Foundation, *State Requirements for the Provision of Medication Abortion*, https://www.kff.org/womens-health-policy/state-indicator/state-requirements-for-the-provision-of-medication-abortion/ (last accessed on Aug. 30, 2022).

217 Ark. Code Ann. § 20–16-606.

218 Iowa Code Ann. § 707.7 (abortion provider must be licensed to practice medicine or osteopathic medicine *and surgery*).

219 Ark. Code Ann. § 20–16-606.

220
*See*  *Mifeprex Patient Agreement Form,*  https.//www.accessdata.fda.gov/drugsatfda_docs/rems/Mifepres_2016-03-29_Patient_Agreement_Form.pdf.

221
*2016 Mifeprex Risk Assessment and Risk Mitigation Reviews* (Mar. 29, 2016) at 2, https://www.accessdata.fda.gov/drugsatfda_docs/nda/2016/020687Orig1s020RiskR.pdf. The reviewers noted that both Planned Parenthood and the National Abortion Federation require patient counseling and informed consent as the standard of care. *Id.* at 8–9.

222
*2016 Mifeprex Risk Assessment and Risk Mitigation Review(s)* at 2 (enclosing *Letter from Janet Woodcock, M.D.* at 1 (Mar. 28, 2016)).

223
*Id.*

224
*See* KRS § 311.7735 (requiring that patient be given extensive information about how to reverse the effects of mifepristone).

225
*GenBioPro*, *supra*, Defendant’s Memorandum in Opposition to Motion for Leave to Amend (‘Mem. Opp.’)(Aug. 4, 2022) at 8–9.

226 Cohen et al*., supra*, at 45.

227
*Id.* at 54*.*

228 Zettler et al*., supra* at 35–36.

229
*Id.* at 37.

230 U.S. Const. Art. 6, cl. 2.

231
*Fidelity Federal Savings and Loan Ass’n v de la Cuesta*, 458 U.S. 141 (1982).

232
*Id*. at 153.

233
*Free v Bland,* 369 U.S. 663, 666 (1962).

234
*Gibbons v Ogden,* 9 Wheat 1 (1824).

235
*Free, supra*, 369 U.S. at 666.

236
*Gade v National Solid Waste Management,* 505 U.S. 88, 108 (1992).

237
*GBP, supra*, Mem. Opp. at 12–13.

238 142 S. Ct. 2587 (2022).

239
*Id*. at 2610–14.

240
*FDA v Brown & Williamson*, 529 U.S. 120 (2000).

241 529 U.S. at 133–42.

242
*Id*. at 142–56. After the Supreme Court ruled that Congress never intended that FDA should regulate cigarettes under the FDCA, Congress fashioned a different statute regulating them not as ‘drugs’ but as a unique product governed by different standards. See 21 U.S.C. § 387b (2021).

243 529 U.S. at 144.

244
*Dobbs, supra*, 142 S. Ct. at 2309 (Kavanaugh, J *concurring*). *See also*  *Id*. at 2277 (*Majority Opinion*) (‘Our decision returns the issue of abortion to those legislative bodies.’).

245 H.R. 5136, 117th Cong., 1st Sess. (Aug. 31, 2021), § 2.

246 H.R. 3755, 117th Cong., 1st Sess. (Sept. 29, 2021), § 4(a)(4), (5), and (7).

247 In the final days of the 117th Congress, the Democratic members (without a single Republican vote) passed a non-binding House Resolution ‘reaffirm[ing] the well-established authority of the Food and Drug Administration to approve, license, grant authorization for, or clear reproductive health products’ and ‘the preemptive effect of those federal laws on any law, requirement, regulation, standard, or other provision enacted, otherwise established, or enforced by any State or political subdivision of a State that prohibits or limits access to or use of any reproductive health product…’ H. Res. 1434 (117th Cong., 2d Sess. Dec. 21, 2022).

248
*Dobbs, supra*, at 2312 (Roberts, C.J., concurring in judgment only).

249
*Dobbs, supra*, at 2265.

